# Structural basis of substrate progression through the bacterial chaperonin cycle

**DOI:** 10.1073/pnas.2308933120

**Published:** 2023-12-08

**Authors:** Scott Gardner, Michele C. Darrow, Natalya Lukoyanova, Konstantinos Thalassinos, Helen R. Saibil

**Affiliations:** ^a^Institute of Structural and Molecular Biology, Department of Biological Sciences, Birkbeck, University of London, London WC1E 7HX, United Kingdom; ^b^SPT Labtech, Melbourn, Cambridge SG8 6HB, United Kingdom; ^c^Division of Biosciences, Institute of Structural and Molecular Biology, University College London, London WC1E 6BT, United Kingdom

**Keywords:** chaperonins, CryoEM, protein folding, Rubisco

## Abstract

The bacterial chaperonin GroEL-GroES promotes protein folding through ATP-regulated cycles of substrate protein binding, encapsulation, and release. Here, we have used cryoEM to determine structures of GroEL, GroEL-ADP·BeF_3_, and GroEL-ADP·AlF_3_-GroES all complexed with the model substrate Rubisco. Our structures provide a series of snapshots that show how the conformation and interactions of non-native Rubisco change as it proceeds through the GroEL-GroES reaction cycle. We observe specific charged and hydrophobic GroEL residues forming strong initial contacts with non-native Rubisco. Binding of ATP or ADP·BeF_3_ to GroEL-Rubisco results in the formation of an intermediate GroEL complex displaying striking asymmetry in the ATP/ADP·BeF_3_-bound ring. In this ring, four GroEL subunits bind Rubisco and the other three are in the GroES-accepting conformation, suggesting how GroEL can recruit GroES without releasing bound substrate. Our cryoEM structures of stalled GroEL-ADP·AlF_3_-Rubisco-GroES complexes show Rubisco folding intermediates interacting with GroEL-GroES via different sets of residues.

## Significance

A central question about the action of molecular chaperones in assisting protein folding in vivo is to understand how a chaperone can provide folding assistance with little or no specificity for substrate sequence or final fold. The bacterial chaperonins GroEL/GroES are among the best understood chaperones, but crucial steps in nucleotide binding and substrate encapsulation have remained obscure. Using cryoEM to examine chaperonin-substrate complexes at different stages along the pathway of assisted protein folding, this study reveals a succession of specific sites of interaction of the substrate with GroEL and provides a structural basis for the central step in chaperonin action—how the non-native substrate is ejected from its hydrophobic binding sites and simultaneously encapsulated in a hydrophilic folding chamber.

Chaperonins prevent protein aggregation and promote correct folding through ATP-driven cycles of binding, encapsulation, and release of substrate proteins (1). The *Escherichia coli* GroEL-GroES system is the archetypical chaperonin and is among the best studied molecular chaperones (2). GroEL subunits assemble into a tetradecameric complex composed of two back-to-back rings that surround a central cavity (3). The cavity is divided into two halves by the disordered C-terminal tails of GroEL subunits. Each GroEL monomer is divided into three domains: the nucleotide-binding equatorial domain, the apical domain that binds GroES and substrate, and an intermediate hinge domain. GroEL binds to non-native proteins through two apical domain helices (helices H and I) that form a hydrophobic collar around the entrance of each GroEL cavity (4). Binding of ATP causes conformational changes in GroEL that facilitate binding of the co-chaperonin GroES to seal the folding chamber (5). The ATP-induced conformational changes of GroEL can also lead to forced unfolding of the bound substrate, which occurs as a result of a stretching force applied to it (6). Forced unfolding of substrate proteins may be necessary to rescue them from kinetically trapped misfolded states and can enhance overall folding rates (7). However, a structural basis for forced unfolding by GroEL has not been described, and our previous cryoEM study of GroEL-ATP was carried out in the absence of a substrate protein (8).

Structural studies of different substrates bound to nucleotide-free GroEL have been published, including malate dehydrogenase (9), gp23 (10), PepQ (11), Rubisco (12), actin (13), and PrP (14). Together, these studies show that non-native proteins initially bind multivalently and in different configurations to GroEL apical domains, allowing GroEL to capture structurally distinct folding intermediates. The GroEL C-terminal tails, while not essential in vivo, also participate in capture and folding of substrate proteins, partly by promoting their deeper initial binding inside the GroEL cavity (15, 16).

Following binding of ATP and the heptameric co-chaperonin GroES, the GroEL-GroES cavity approximately doubles in volume and in principle can accommodate substrate proteins up to a mass of around 60 to 70 kDa (17). The majority of GroEL substrates identified in *E. coli* are 20 to 40 kDa, with a sharp cutoff toward proteins above 50 kDa (18). Two published cryoEM reconstructions of *Rhodospirillum rubrum* Rubisco (50.5 kDa) encapsulated by GroEL-GroES show either a native-like density (19) or a non-native-like density (15) located in the lower part of the cavity, interacting with hydrophobic residues of the cavity wall.

To gain insights into GroEL-assisted protein folding, we used cryoEM to determine structures of GroEL, GroEL-ADP·BeF_3_, and GroEL-ADP·AlF_3_-GroES, each complexed with the obligate substrate *R. rubrum* Rubisco. Our reconstruction of nucleotide-free GroEL-Rubisco shows that Rubisco fills the GroEL cavity and interacts with several GroEL apical domains and C-terminal tails. By studying GroEL-ADP·BeF_3_- Rubisco, we identified an asymmetric conformation of the substrate-bound GroEL ring. Four GroEL subunits maintain contact with non-native Rubisco while the remaining three subunits extend upward. In addition, we observed a more extensive interaction between the substrate and the GroEL C termini. This complex offers a possible mechanism for forced unfolding in which the substrate protein is stretched between the apical domains and C termini of GroEL subunits while other GroEL subunits simultaneously present sites for GroES binding. Upon recruitment of GroES, Rubisco is encapsulated in the folding chamber where it samples non-native and native-like conformations, held in place by interactions with hydrophobic and charged residues of GroEL-GroES.

## Results

### CryoEM Structure of GroEL Bound to Non-Native Rubisco.

Protein folding intermediates can be captured by rapidly diluting chemically denatured substrate proteins into a GroEL-containing buffer (20). We formed binary complexes of wild-type *E. coli* GroEL bound to the model substrate protein *R. Rubrum* Rubisco. We confirmed the formation of binary complexes using native gel electrophoresis and native mass spectrometry (*SI Appendix*, Fig. S1).

Our initial attempts to determine a cryoEM reconstruction of GroEL-Rubisco were hindered by preferred orientation (*SI Appendix*, Fig. S2). In the absence of non-native substrate, GroEL particles adopted a range of orientations permitting high-resolution refinement (*SI Appendix*, Fig. S2*A*). However, particles of GroEL bound to non-native Rubisco exhibited a strongly preferred end-view orientation. This limited the resolution of the reconstruction, and non-native Rubisco was not well resolved (*SI Appendix*, Fig. S2*B*). To offset the preferred orientation, we collected cryoEM data employing stage tilt (*SI Appendix*, Fig. S2*C*). Despite the lower number of particles, density for non-native Rubisco was better resolved (*SI Appendix*, Fig. S2*C*).

To attain a higher resolution reconstruction, we aimed to reduce the interaction of GroEL-Rubisco with the air-water interface during cryoEM grid preparation. To accomplish this we prepared cryoEM grids of GroEL-Rubisco using a Chameleon (21, 22). Chameleon dispenses liquid sample onto a self-wicking grid, then after a short wicking time, plunge freezes the grid into liquid ethane. We collected cryoEM data of GroEL-Rubisco from two grids and determined a reconstruction from each. Although there was still preferred orientation, enough alternate views were recorded to yield isotropic reconstructions (*SI Appendix*, Fig. S3*A*). We used 3D classification to separate apo GroEL particles from GroEL-Rubisco particles, and then combined GroEL-Rubisco from each dataset (*SI Appendix*, Fig. S4).

**Fig. 1.**
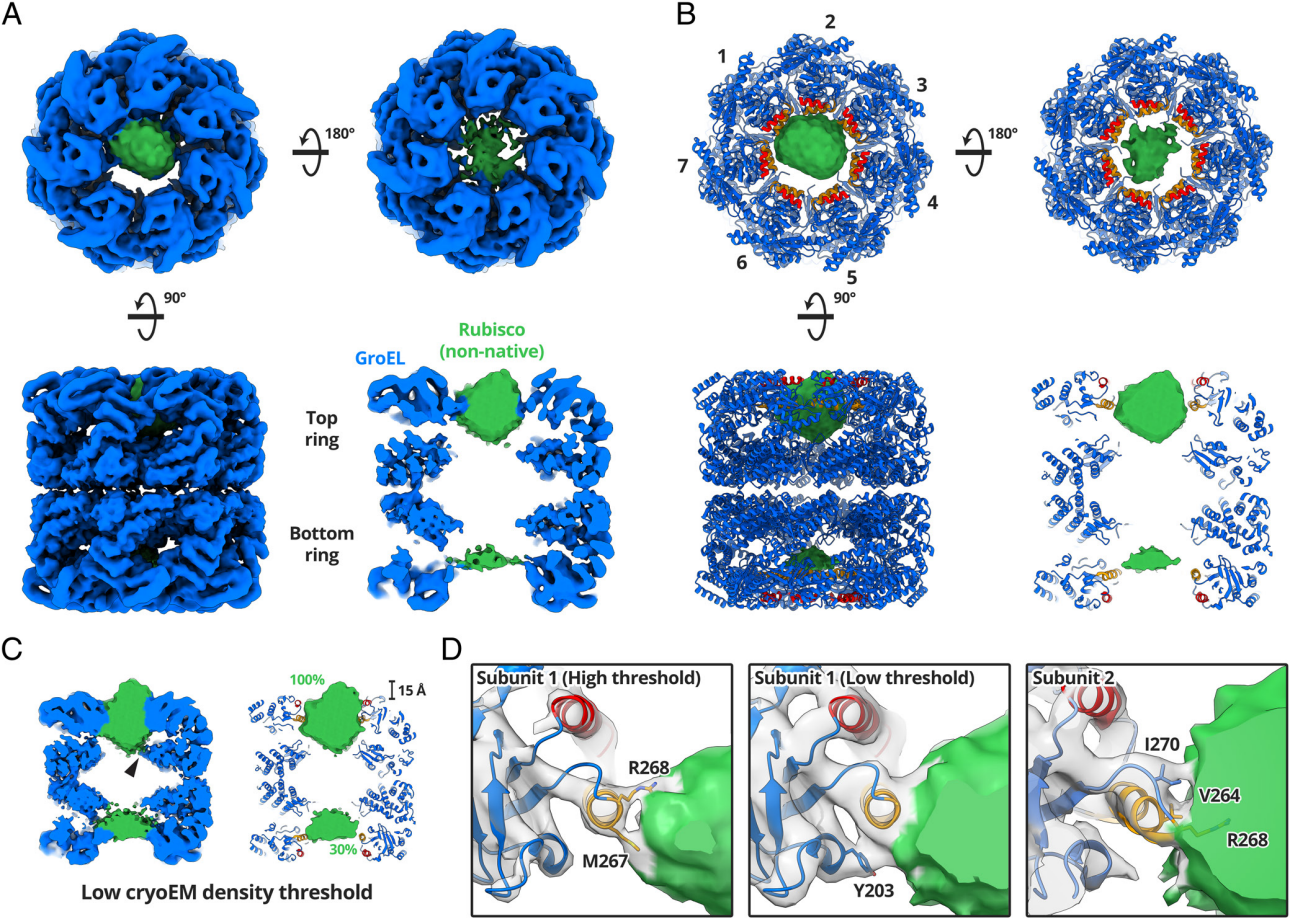
CryoEM structure of GroEL-Rubisco. (*A*) CryoEM map of GroEL-Rubisco at 4.5 Å. CryoEM density is shown coloured blue (GroEL) and green (Rubisco). (*B*) Refined atomic model of GroEL and Rubisco density (green) contoured at 8.0σ. The atomic model of GroEL is coloured blue; the substrate-binding helices H and I are coloured red and orange, respectively. (*C*) CryoEM map of GroEL-Rubisco contoured at a low threshold (5.0σ). The black arrowhead indicates a possible interaction between non-native Rubisco and the GroEL C termini. Percent values in green text represent the Rubisco density compared to that of a folded Rubisco monomer. (*D*) Contacts between GroEL subunits 1, 2, and 3 (gray density), and non-native Rubisco (green density). Interacting GroEL residues are labelled and shown as stick models.

A 4.4 Å cryoEM map of GroEL-Rubisco was reconstructed from 65,453 particles (Fig. 1*A* and *SI Appendix*, Figs. S3*A* and S4 and Table S1). The local resolution of the map ranged from 4.2 Å for GroEL equatorial domains to worse than 12 Å for non-native Rubisco (*SI Appendix*, Fig. S3*A*). We refined the atomic model of GroEL (PDB code: 1SS8) into the cryoEM map (Fig. 1*B*). Extra density inside the GroEL cavities was attributed to bound non-native Rubisco. At a moderate contour level (5.0σ), Rubisco density in the top ring represented the full volume estimated for a folded Rubisco monomer (~61,000 Å^3^) (Fig. 1*C*). Density for non-native Rubisco was also present in the bottom GroEL ring (Fig. 1 *A*–*C*). However, this density was weaker and represented only around 30% of the volume of a folded Rubisco monomer (Fig. 1*C*). For comparison, the Rubisco density in reconstructions from our initial cryoEM datasets accounted for 20 to 50% of a natively folded monomer (*SI Appendix*, Fig. S2).

Non-native Rubisco was positioned at the level of the GroEL apical domains (Fig. 1*B*). When displayed at a high contour level (8.0σ), the map revealed individual contacts between non-native Rubisco and the apical domains of three GroEL subunits. Several additional features of the complex were apparent at lower map contour levels (5.0σ) (Fig. 1*C*). At these lower contour levels, Rubisco density filled the top GroEL cavity and contacted all seven GroEL subunits via helix H, helix I, and the underlying hydrophobic segment. As the contour level is lowered further (<5.0σ), the density for non-native Rubisco and several of the GroEL C termini becomes continuous, this might suggest a weak interaction. (Fig. 1*C*, black arrow). The Rubisco density also protruded ~15 Å above the level of helix H (Fig. 1*C*).

### Interactions between GroEL and Non-Native Rubisco.

By thresholding the map through a range of contour levels (5 to 12σ), we identified specific GroEL residues involved in binding non-native Rubisco (Fig. 1*D*). The strongest contacts to Rubisco were in helix I, consistent with previous cryoEM studies of different GroEL-substrate complexes (9–14). Many of the residues involved in contacting non-native Rubisco were those identified in the original mutational studies of GroEL (4). These included V264 and Y203, both implicated in substrate binding and located in helix I and the underlying hydrophobic segment, respectively (4). However, we also identified substrate-binding residues located at the C-terminal end of helix I, including M267, R268, and I270. The relative importance of these residues is less clear. GroEL-substrate interactions are canonically hydrophobic, explaining the contacts observed for M267 and I270. M267 has been implicated in allosteric communication, though an exact role is not known (23). Perhaps most surprising is the interaction with the positively charged residue R268, which was consistently the strongest interacting residue in our cryoEM reconstructions. GroEL R268 has previously been shown to form hydrogen bonds to glycine and serine residues on a 12-residue GroEL-binding peptide (24).

### ATP Binding Induces Asymmetry in the Rubisco-Bound Ring of GroEL.

We next aimed to study the effects of ATP binding to GroEL-Rubisco, building on our previous work on GroEL-ATP (8). GroEL-Rubisco complexes were prepared as described above, then blotted and plunge-frozen with a Vitrobot several seconds after addition of ATP. We collected cryoEM data employing stage tilt (25) to compensate for the preferred orientation of GroEL-ATP-Rubisco (*SI Appendix*, Fig. S3*B*). Initial reconstructions showed that GroEL had partially denatured at the air-water interface (*SI Appendix*, Fig. S5). We used a combination of signal subtraction and 3D classification to identify a subset of 13,015 relatively undamaged particles (*SI Appendix*, Fig. S5). We determined a reconstruction of GroEL-ATP at a resolution of 4.3 Å (*SI Appendix*, Fig. S5). The map showed an asymmetric ring arrangement of GroEL. However, the low resolution limited interpretability. We could not reliably identify bound nucleotide, and density for non-native Rubisco was not well resolved.

For high-resolution cryoEM, we replaced ATP with a nonhydrolysable analogue. The ADP-metal complexes ADP·BeF_3_, ADP·AlF_3_, and ADP.VO_4_ are mimics of the ATP ground state, transition state, and posthydrolysis state, respectively (26). Incubation of GroEL-GroES with ADP + BeF_3_ or ATP + BeF_3_ supports formation of asymmetric GroELGroES_1_ and symmetric GroEL-GroES_2_ complexes, respectively (27). Both ADP·BeF_3_ and ADP·AlF_3_ support folding of the GroEL substrate Rhodanese in the presence of GroES (26). Both ATP analogues have been used to aid previous structural studies of chaperonins (10, 19, 28). Additionally, to reduce both preferred orientation and denaturation at the air-water interface, we used the Chameleon instrument to prepare grids for cryoEM.

**Fig. 2.**
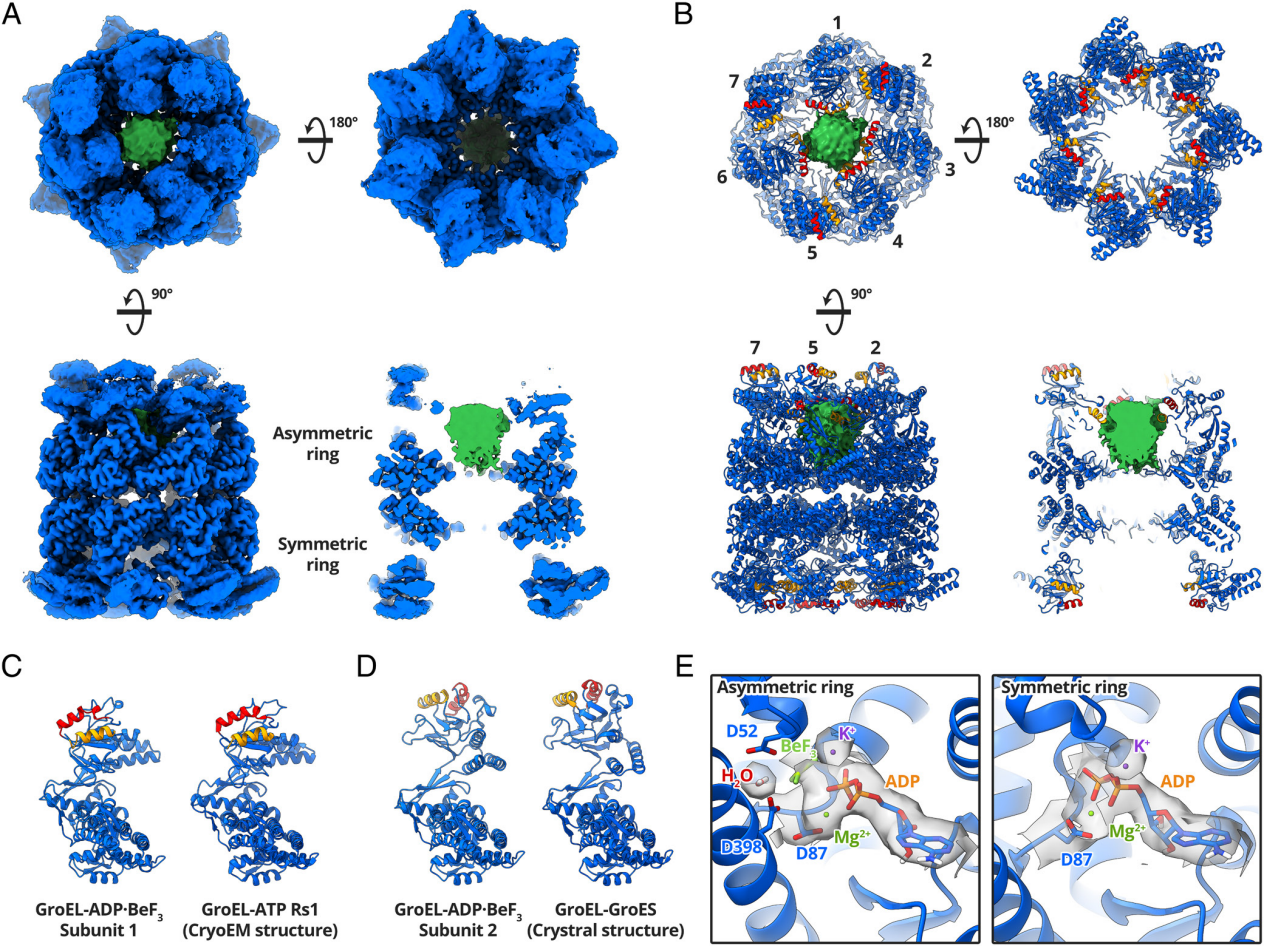
CryoEM structure of GroEL-ADP·BeF_3_-Rubisco. (*A*) CryoEM map of GroEL-ADP·BeF_3_-Rubisco at 3.4 Å. The GroEL map (blue) displayed was generated by DeepEMhancer. Density for non-native Rubisco (green) was isolated from the locally filtered map generated by Relion. (*B*) Refined atomic model of GroEL-ADP·BeF_3_ and non-native Rubisco density (green). The substrate-binding helices H and I are coloured red and orange, respectively. The asymmetry can be appreciated from the position of helix H in each subunit. (*C*) Comparison of GroEL-ADP·BeF_3_ subunit 1 with the published structure of the Rs1 conformation of GroEL-ATP (PDB: 4AAQ). (*D*) Comparison of GroEL-ADP·BeF_3_ subunit 2 with the published crystal structure of GroEL-GroES (PDB: 1SVT). (*E*) Nucleotide binding sites of each GroEL ring, showing ADP·BeF_3_ in asymmetric ring subunits and ADP in symmetric ring subunits. Overlaid cryoEM density is shown only for the labelled moieties.

A 3.4 Å cryoEM map of GroEL-ADP·BeF_3_-Rubisco was reconstructed from 202,582 particles (Fig. 2 and *SI Appendix*, Figs. S3*C* and S6 and Table S1). The map displayed an asymmetric ring and a symmetric ring (Fig. 2*A*). We observed density for Rubisco in the asymmetric ring only, contacting four GroEL subunits (Fig. 2*A*). The apical domains of the remaining three GroEL subunits were less well resolved and they extended upward, adopting a conformation reminiscent of the GroES- bound state (Fig. 2*A*). We used DeepEMhancer (29) to visualise the extended GroEL apical domains. However, Rubisco density was absent from the DeepEMhancer map, likely due to low local resolution. We used the locally filtered map from Relion to build a model of the complex and used the DeepEMhancer map only to position the apical domains of GroEL subunits 2, 5, and 7. We built and refined the model into the cryoEM maps using the crystal structures of apo GroEL (PDB code: 1SS8) and GroEL-GroES (PDB: 1SVT) (Fig. 2*B*). The conformation of the four substrate-contacting GroEL subunits resembled the Rs1 conformation previously reported for GroEL-ATP (Fig. 2*C*). In the Rs1 state, the GroEL intermediate and apical domains have undergone a 35° sideways tilt as a single rigid body relative to the nucleotide-free state (8). The four Rs1 GroEL subunits shared the equatorial-to-apical domain salt bridge, R58-E209, not observed in our previous study of GroEL-ATP (*SI Appendix*, Fig. S3*D*). R58 is located within a short α-helix adjacent to the stem loop of GroEL equatorial domains. E209 lies in a short loop region of the underlying hydrophobic segment in the apical domains. In both the 2.7 Å crystal structure of apo GroEL (PDB: 1SS8) and in our 4.4 Å cryoEM reconstruction of nucleotide-free GroEL-Rubisco, the E209 loop faces away from the R58 helix and the R58-E209 sidechains are ~8 Å apart (*SI Appendix*, Fig. S3*D*). This salt bridge likely only forms upon binding of ATP (or analogue) and may act to stabilise the substrate-bound Rs1 state. The salt bridge could also be involved in allosteric communication between the apical and equatorial domains of GroEL. The residue E209 is located adjacent to the underlying hydrophobic segment which is involved in substrate-binding primarily via Y203. GroEL subunits 2, 5, and 7 adopted the GroES-bound state (Fig. 2*D*). This GroEL subunit conformation has only previously been observed in structures of GroEL-GroES, never in the absence of GroES. Our structure of GroEL-ADP· BeF_3_-Rubisco likely represents a transient intermediate complex adopted in response to ATP and substrate binding. This conformation of GroEL might be able to recruit GroES without releasing non-native substrate, and potentially represents a missing link in substrate encapsulation.

We examined the nucleotide binding sites of GroEL subunits (Fig. 2*E*). Clear density for ADP was seen in all fourteen sites. We observed differences in nucleotide site density between the two rings, but saw no obvious differences among subunits belonging to the same ring (*SI Appendix*, Fig. S3*E*). We observed continuous density between the GroEL D87 sidechain and ADP. D87 is involved in ATP hydrolysis, and mutations such as D87K abolish ATPase activity (4). We were able to confidently model ADP and the phosphate oxygen-coordinating metal, Mg^2+^. ADP bound in the asymmetric ring showed additional density that we attributed to the ATP γ-phosphate analogue, BeF_3_. (Fig. 2*E*). Symmetric ring ADP lacked this additional density and it was modelled without BeF_3_. Subunits in both rings showed density for the second coordinating metal ion, K^+^. GroEL requires K^+^ to hydrolyse ATP, and a previously published crystal structure confirmed this position as the K^+^ binding site (30). In the asymmetric ring, we observed additional density between the D52 and D398 sidechains (Fig. 2*E*). We attributed this to the water molecule involved in attacking the γ-phosphate of ATP during hydrolysis (31). Asymmetric ring subunits have therefore been captured in an ATP-bound state prior to hydrolysis, consistent with the classification of ADP·BeF_3_ as a ground-state analogue of ATP.

### Interactions between Non-Native RuBisCO and GroEL-ADP·BeF_3_.

**Fig. 3.**
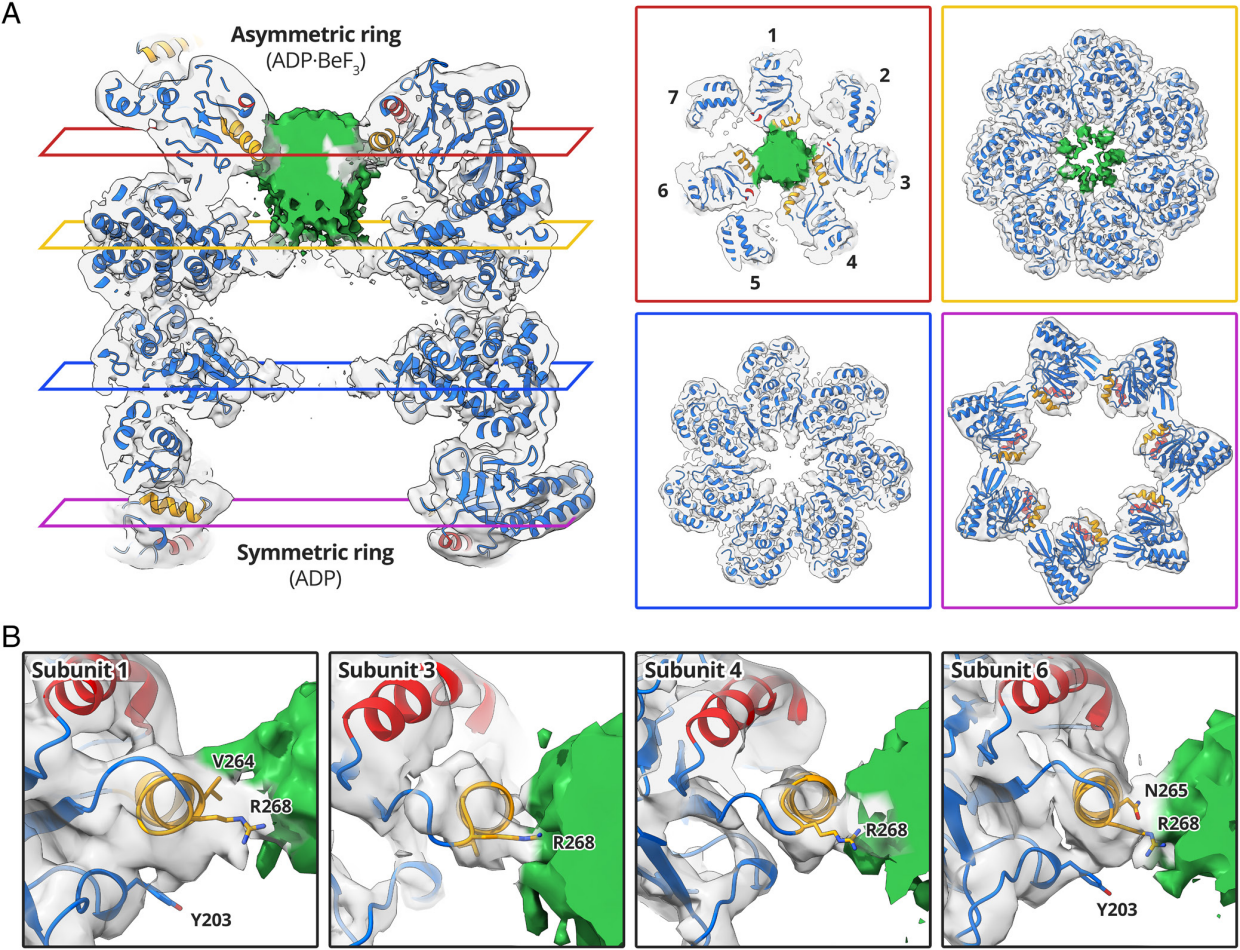
Interactions between GroEL-ADP·BeF_3_ and non-native Rubisco. (*A*) Central slices through the GroEL-ADP·BeF_3_-Rubisco model overlaid with the cryoEM map. GroEL density is coloured transparent gray; Rubisco/GroEL C-terminal density is coloured green. Panels showing lateral slices through the asymmetric ring apical domains (red panel), asymmetric ring equatorial domains (yellow panel), symmetric ring equatorial domains (blue panel), and symmetric ring apical domains (purple panel). (*B*) Interactions between GroEL apical domains and non-native Rubisco.

Non-native Rubisco interacted with the apical domains of four GroEL subunits in the asymmetric ring (Fig. 3). At low contour levels, the interaction was dominated by helix I and the underlying hydrophobic segment of GroEL subunits 1, 3, 4, and 6 (Fig. 3*A*), leaving subunits 2, 5, and 7 to extend upward. The density attributed to Rubisco extended deeper into the GroEL cavity (Fig. 3*A*) than in our reconstruction of nucleotide-free GroEL-Rubisco (Fig. 1). This raises the possibility that part of the density instead represents the seven C termini of GroEL, which together have a mass of 14 kDa. If the lower part of the density inside the GroEL cavity represents the C termini, it suggests a direct interaction with non-native Rubisco in the upper cavity. At a moderate contour level (5.0σ), the volume of the Rubisco/GroEL C-terminal density in the asymmetric ring accounted for a mass of ~34 kDa. In the symmetric ring, we observed density for the C-terminal GroEL residues P525 and K526 (typically disordered in crystal structures), but no density for non-native Rubisco (Fig. 3*A*). We examined the contacts between GroEL apical domains and non-native Rubisco at higher contour levels (Fig. 3*B*). The strongest interactions were similar to those we observed in the nucleotide-free binary complex (Fig. 1). The same R268 contact in helix I was seen for each of the four GroEL subunits (Fig. 3*B*). Other subunits showed contacts involving V264 and N265 of helix I, and residue Y203 of the underlying hydrophobic segment (Fig. 3*B*).

### Rubisco Encapsulated by GroEL-ADP·AlF_3_-GroES.

**Fig. 4.**
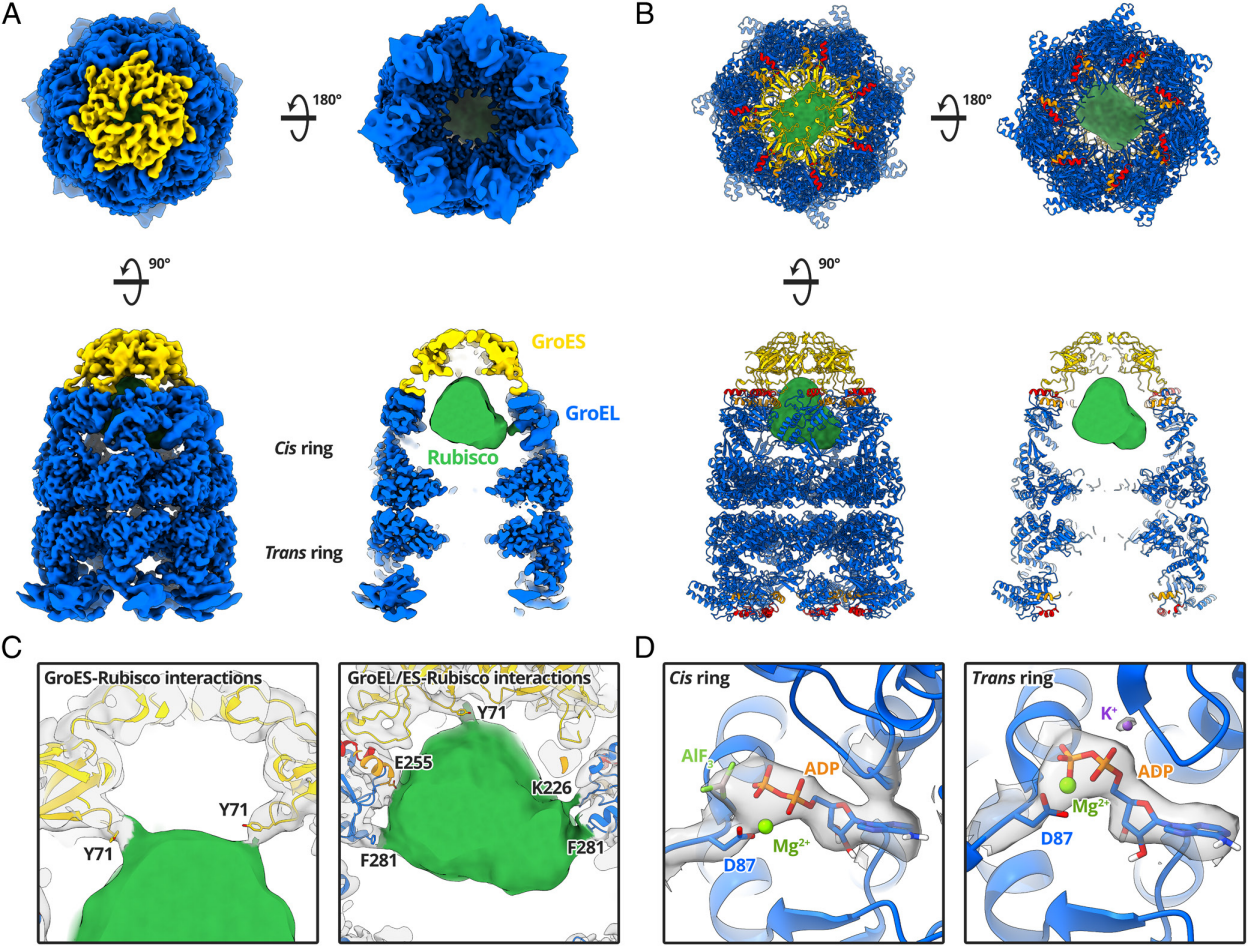
CryoEM structure of GroEL-ADP·AlF_3_-Rubisco-GroES. (*A*) CryoEM map of GroEL-ADP·AlF_3_-Rubisco-GroES at a global resolution of 3.7 Å, filtered by local resolution. (*B*) Refined atomic model of GroEL-ADP·AlF_3_-GroES and non-native Rubisco density (green). (*C*) Molecular contacts between GroEL-GroES and Rubisco. (*D*) Nucleotide site density in the *cis* and *trans* rings.

We next studied the conformation of encapsulated Rubisco in the full GroEL-GroES complex. We added GroES and ADP·AlF_3_ to GroEL-Rubisco to form stalled ternary complexes. We again used a Chameleon instrument to prepare frozen grids. Initial 3D classification showed variability in the occupancy of GroES (*SI Appendix*, Fig. S7). Two of the 3D classes showed GroEL-ADP·AlF_3_ complexes without GroES. We processed these classes and determined a 3.7 Å structure of GroEL-ADP·AlF_3_-Rubisco, which in the absence of GroES, displayed the same asymmetric conformation observed for GroEL-ADP·BeF_3_-Rubisco (*SI Appendix*, Fig. S8). To identify GroEL-GroES particles with encapsulated Rubisco, we used masked 3D classification targeting the *cis* cavity (*SI Appendix*, Fig. S7). We determined a 3.7 Å reconstruction of GroEL-ADP·AlF_3_-Rubisco-GroES from 30,965 particles (Fig. 4*A* and *SI Appendix*, Figs. S3*F* and S7 and Table S1). We refined the published crystal structure of GroEL-GroES (PDB: 1SVT) into our cryoEM map (Fig. 4*B*). Density for encapsulated Rubisco occupied the upper two-thirds of the *cis* cavity, adjacent to the GroEL apical domains. The Rubisco density accounted for 40 to 50 kDa of protein mass, and its shape was reminiscent of a folded Rubisco monomer. Interactions were observed with several cavity-facing residues of GroEL-GroES subunits (Fig. 4*C*). The strongest contacts to encapsulated Rubisco involved GroEL residue F281, and GroES residue Y71 (Fig. 4*C*). The ring of Y71 residues on GroES subunits forms a hydrophobic collar on the ceiling of the cis cavity and may be important for the folding of some GroEL substrates (32). Additional contacts were resolved at lower map contour levels and involved GroEL residues K226 and E255 (Fig. 4 *C*, *Right*).

The conformation of the *trans* ring of GroEL-ADP·AlF_3_-Rubisco- GroES resembled the symmetric ring of GroEL-ADP·BeF_3_-Rubisco. This “wide” conformation of the GroEL *trans* ring is likely related to the presence of high concentrations of ADP (3 mM) during sample preparation (33). We did not observe density for non-native Rubisco in the *trans* ring, even at low map contour levels. In this conformation, the continuous hydrophobic collar formed by helices H and I is disrupted, possibly leading to reduced substrate binding. Lower concentrations of ADP may have allowed for visualisation of bound non-native substrate in the *trans* ring.

We observed clear density for ADP in all fourteen nucleotide binding sites. *Cis* ring ADP showed additional density that we modelled as AlF_3_ (Fig. 4*D*). *Trans* ring sites contained the coordinating potassium ion (Fig. 4*D*). At this point along the ATP hydrolysis reaction coordinate, K^+^ has presumably fulfilled its catalytic role and is no longer required in the *cis* ring. In contrast, our structure of GroEL-ADP·BeF_3_-Rubisco showed K^+^ bound in both GroEL rings. This is consistent with BeF_3_ and AlF_3_ mimicking different states of the ATP γ-phosphate.

### Further 3D Classification Revealed Distinct Conformations of Encapsulated Rubisco.

**Fig. 5.**
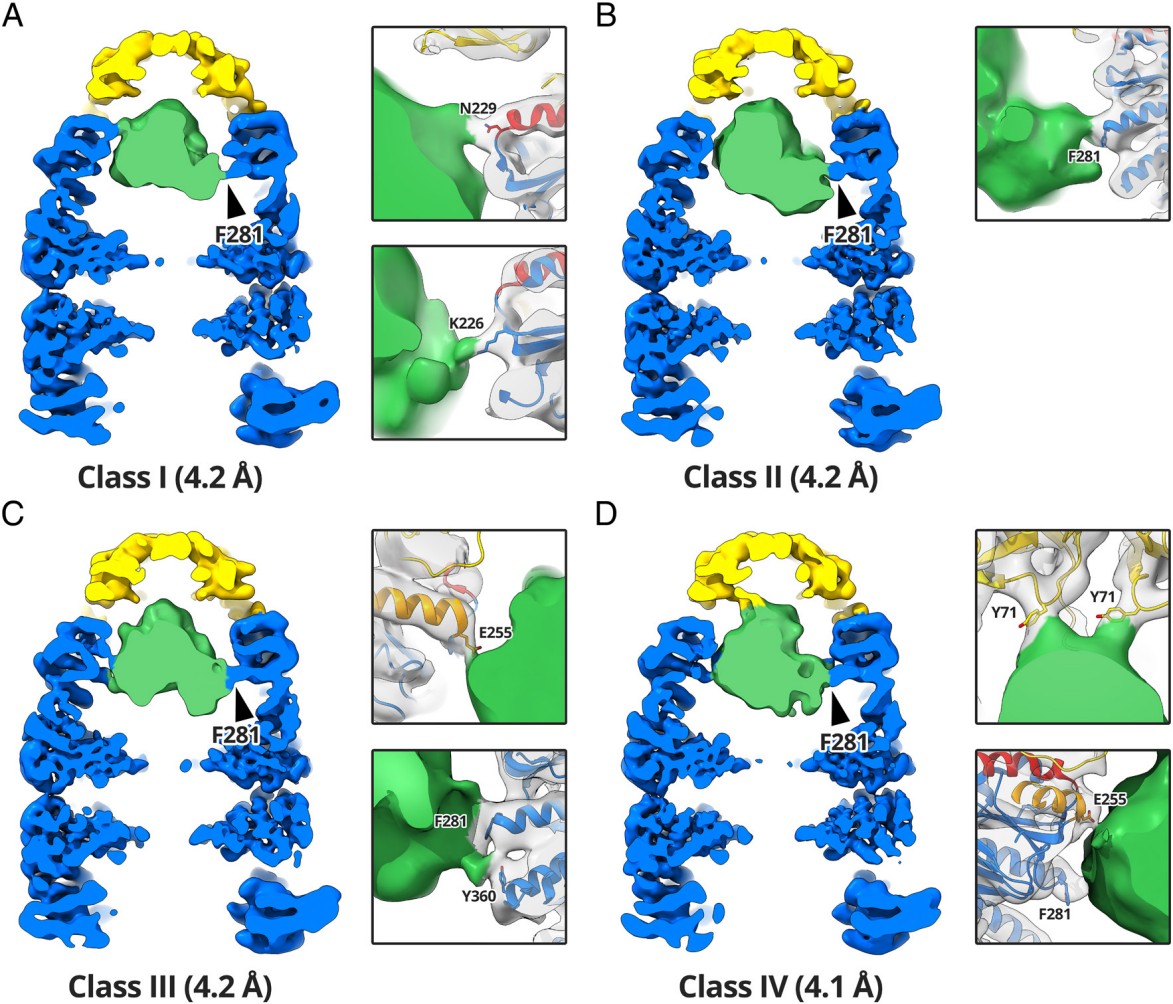
Multiple classes of encapsulated Rubisco. Red dashed circles highlight the contact in all four reconstructions between GroEL residue F281 and Rubisco. (*A*) Reconstruction of class I from 7,202 particles. Panels highlight the K226 and N229 contacts. (*B*) Reconstruction of class II from 8,237 particles. Panel highlights the F281 contact. (*C*) Reconstruction of class III from 7,818 particles. Panels highlight the F281, Y360, and E255 contacts. (*D*) Reconstruction of class IV from 7,708 particles. Panels highlight the E255, F281, and GroES Y71 contacts.

The Rubisco density in our reconstruction of the stalled ternary GroEL-ADP·AlF_3_-Rubisco-GroES complex likely represented an ensemble of conformations that had been averaged together during image processing. We aimed to identify some of these conformations using an additional round of 3D classification (*SI Appendix*, Fig. S7). Due to the relatively low number of particles at this processing step, we opted to use four classes for masked 3D classification, targeting the GroEL-GroES *cis* cavity. We refined each subset of particles to a resolution of 4.1 to 4.2 Å (*SI Appendix*, Fig. S3*G*) and performed a rigid body fit of our refined model of GroEL-ADP·AlF_3_-GroES (Fig. 5). In the four reconstructions the interactions between GroEL-GroES and the encapsulated Rubisco monomer were well resolved. Each class showed a different set of GroEL-GroES residues interacting with Rubisco, suggesting that GroEL-GroES can stabilise a range of non-native substrate conformations (Fig. 5). At lower contour levels, the substrate density accounted for the volume a full Rubisco monomer (~61,000 Å^3^). All four classes shared the same GroEL F281 contact to Rubisco (Fig. 5 *A*–*D*, black arrowheads). Individual classes displayed additional contacts from the Rubisco density to GroEL residues K226 (Fig. 5*A*), N229 (Fig. 5*A*), E255 (Fig. 5 *C* and *D*), and Y360 (Fig. 5*C*). Additionally, class 4 displayed strong contacts to the Y71 residues of two adjacent GroES subunits (Fig. 5*D*).

### Model of Near-Native Rubisco Encapsulated in the GroEL-GroES Folding Chamber.

**Fig. 6.**
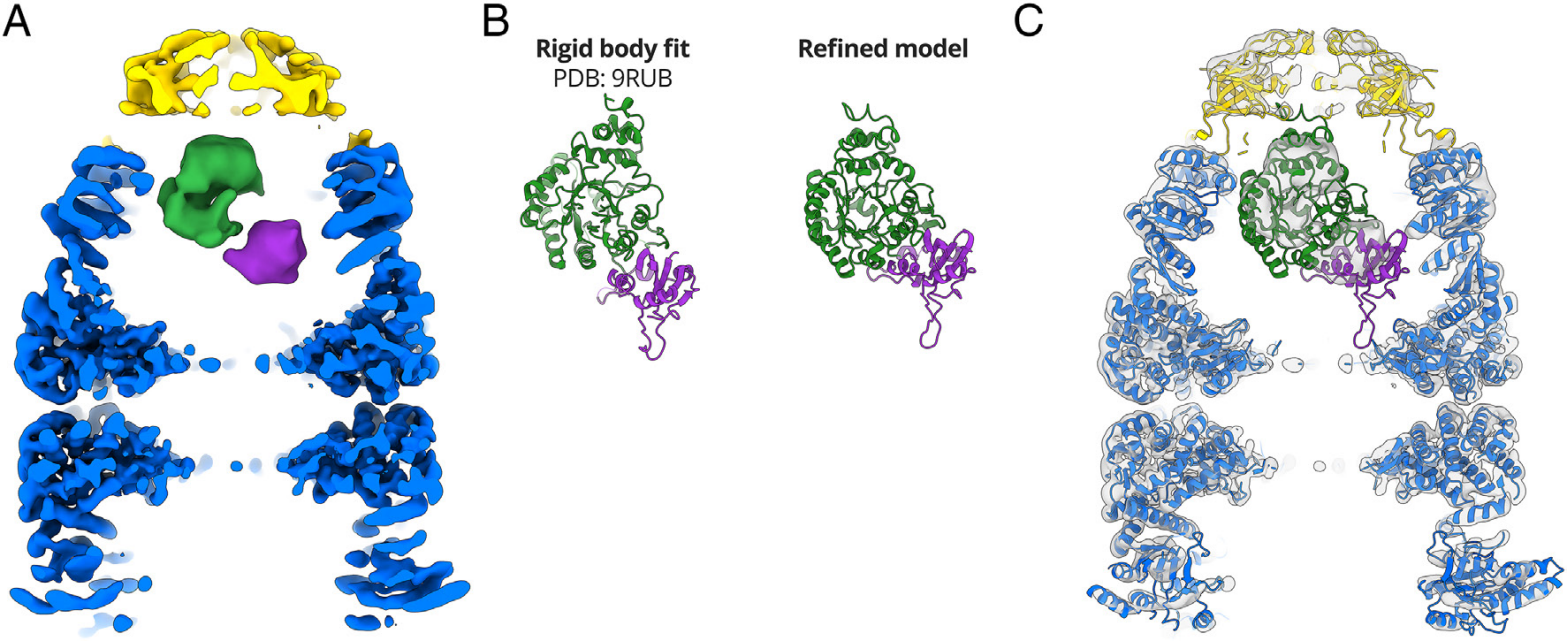
Modelling Rubisco inside the GroEL-GroES folding chamber. (*A*) CryoEM map of GroEL-ADP·AlF_3_-Rubisco-GroES (class II) at a contour level of 7σ. The two domains of the encapsulated Rubisco monomer are coloured purple (NTD) and green (CTD). (*B*) Comparison between the crystal structure of a Rubisco monomer and the refined model. (*C*) Refined model of GroEL-ADP·AlF_3_-Rubisco-GroES overlaid on the class II density at a contour level of 3σ.

To model Rubisco, we examined the density in the four cryoEM reconstructions. Due to the low local resolution, we were unable to identify secondary structure elements of Rubisco. We limited our analysis to low-resolution features and examined density that might represent the different Rubisco domains. Rubisco monomers are composed of two domains, an N-terminal domain (NTD; residues 1 to 135) and a larger C-terminal TIM barrel domain (CTD; residues 136 to 466) (34). Classes I, III, and IV could accommodate rigid body fits of the Rubisco monomer in multiple different orientations. The class II reconstruction (*SI Appendix*, Fig. S7) showed two distinct lobes of density when displayed at a higher contour level (Fig. 6*A*). We attributed these lobes to the NTD and CTD of Rubisco and used them to orient and rigid body fit the published Rubisco crystal structure (PDB: 9RUB) (Fig. 6*B*). We flexibly fit the Rubisco monomer into the density, allowing for only minor changes when optimising the map-model fit (Fig. 6 *B* and *C* and *SI Appendix*, Table S1).

## Discussion

In this study, we have used single-particle cryoEM to determine structures GroEL, GroEL-ADP·BeF_3_, and GroEL-ADP-AlF_3_-GroES all complexed with non-native Rubisco. Our work provides a series of snapshots of chaperonin complexes with a non-native protein as it progresses through the GroEL-GroES reaction cycle, revealing the interactions between GroEL and substrate at each step. We have described a conformation of ATP-bound GroEL that can simultaneously recruit its co-chaperonin GroES while still binding non-native substrate, preventing its escape during the encapsulation step. Lastly, we showed that encapsulated Rubisco resides in the GroEL-GroES cavity as an ensemble of conformational states that likely represent different folding intermediates.

We have previously shown that Rubisco binds to the apical domains of GroEL subunits (12) in a similar fashion to other GroEL substrates (9–11, 13, 14). Structural studies of GroEL-substrate complexes are typically limited to low resolution and density for non-native substrate is usually incomplete. Our cryoEM reconstruction of GroEL-Rubisco showed that the strongest contacts were formed with helix I of GroEL, consistent with previous structural studies of other GroEL-substrate complexes. Several of the residues involved in contacting non-native Rubisco were those identified in the original mutational studies of GroEL, such as V264 and Y203 (4). Recognition of substrates by GroEL is typically described as predominantly hydrophobic, involving nonpolar residues. We therefore did not expect the strongest contact to Rubisco to involve the GroEL residue R268, located at the C terminus of helix I. The importance of R268 in substrate binding and folding is less well characterised. Structural evidence for the role of R268 in mediating substrate interactions comes from the crystal structure of GroEL bound to a 12-residue peptide (24). The GroEL-peptide structure showed that a serine and glycine residue on the peptide formed hydrogen bonds to R268 (24). Our cryoEM structures of GroEL-Rubisco and GroEL-ADP·BeF_3_-Rubisco identified R268 as an important residue in mediating non-native substrate interactions prior to GroES binding. Additionally, we observed a strong interaction between non-native Rubisco and GroEL residue M267, located on the lower face of helix I, suggesting that it may play a role in substrate binding. It has been implicated in intra- and intersubunit allosteric communication (23).

Experiments using native mass spectrometry have previously shown that Rubisco monomers bind to GroEL tetradecamers with a 1:1 stoichiometry, exerting negative cooperativity on the opposite GroEL ring and inhibiting binding of a second Rubisco monomer (35). Other substrates with a range of molecular weights (32 to 56 kDa) have been shown to bind to both GroEL rings simultaneously, implying that GroEL can recognise and respond to different types of substrate (36). Our native mass spectrometry results for GroEL-Rubisco agreed with the published results. However, our cryoEM reconstruction showed Rubisco bound in both rings simultaneously, albeit with different occupancies. Previous work has suggested that the structural basis for this negative cooperativity lies in a narrowing of the opposite GroEL ring (12, 15, 36). However, we did not observe a significant structural change in the opposite ring.

All previously published cryoEM structures of GroEL-substrate complexes were determined from grids prepared using conventional plunge-freezing methods that included a blotting step, and a several-second delay between sample application and vitrification. It has been shown that reducing this delay can reduce denaturation at the air-water interface and improve the orientation distribution of particles (22). Previous reconstructions of GroEL-substrate complexes did not display the full expected volume of the non-native substrate. For example, previous studies report the following percentages for the substrate volume in their reconstruction: GroEL-MDH: 25 to 40% (9), GroEL-gp23: 54% (10), GroEL-actin: 28% (13), and GroEL-Rubisco: 30 to 35% (12). Our initial cryoEM attempts using traditional vitrification methods yielded similar results, but our reconstruction from Chameleon grids accounted for the full volume of Rubisco. It is likely that non-native substrate in previous studies had been partially denatured at the air-water interface. The missing density in the published reconstructions would have presumably protruded from the GroEL cavity, as displayed in our reconstruction of GroEL-Rubisco. Non-native proteins are particularly prone to adsorption at the air-water interface during cryoEM grid preparation. Our work shows that reducing the time between sample application and vitrification provides additional benefits in the study of biological systems involving non-native proteins.

Binding of ATP is known to trigger conformational changes within GroEL subunits (8, 37). Studies of GroEL-GroES bound to ATP analogues such as ATPγS or AMP-PNP fail to form folding active complexes and do not show the same large-scale conformational changes in GroEL (26, 38, 39). This is likely related to the critical role of the ATP γ-phosphate, mimicked in our structures by BeF_3_ or AlF_3_ (26). Here, we present the structure of a GroEL intermediate with both nucleotide and substrate bound.

Our previous structural study of GroEL-ATP was carried out in the absence of substrate protein, and the dataset was not large enough to test for asymmetry, particularly in the more open states (8). In this work, we show that individual GroEL subunits of the ATP-bound ring adopt one of two conformations, resulting in a markedly asymmetric ring. This asymmetric behaviour of subunits is reminiscent of the eukaryotic hetero-oligomeric group II chaperonin TRiC/CCT (40). Our cryoEM structure of GroEL-ADP· BeF_3_-Rubisco reveals how some GroEL subunits can recruit GroES while others are bound to non-native substrate, preventing its escape. The four substrate-bound GroEL subunits adopt the Rs1 state reported for GroEL-ATP (8). The three remaining subunits adopt the GroES-bound conformation (41), despite the absence of GroES itself.

In our cryoEM map of GroEL-ADP·BeF_3_-Rubisco, the C-terminal tails of all seven asymmetric ring subunits contacted non-native Rubisco. In comparison, structures of nucleotide-free GroEL-substrate complexes do not typically suggest an extensive interaction with the C termini. This suggests that deeper substrate- binding role of the GroEL C termini becomes more important following ATP binding. Deletion of the GroEL C termini has been shown to slow folding of Rubisco (16). Nevertheless, the C termini themselves are not essential in vivo (42) despite being conserved among chaperonins. The slowed rate of folding upon C-terminal tail deletion has been attributed to altered rates of chaperonin cycling and ATPase activity (discussed in appendix 1 of the comprehensive review of chaperonins in ref. 2).

We previously speculated that GroES is initially recruited by 1 to 2 raised GroEL subunits, resulting in an asymmetric intermediate (8, 9). However, significant asymmetry of a GroEL ring has only been previously reported in the crystal structure of the double mutant GroEL_ΔD83A/ΔR197A_ bound to ADP (43). In this mutant, two intersubunit salt bridges were removed, effectively detaching adjacent apical domains. The freed apical domains adopted conformations similar to those observed for GroEL-ATP (8). Our structure of GroEL-ADP·BeF_3_-Rubisco offers a view of an asymmetric wild-type GroEL ring in an intermediate state of the folding reaction.

During transition from the Rs1 state to the GroES-bound state, GroEL apical domains undergo a dramatic upward swing of 60°, and a 90° clockwise rotation (8). These movements have been suggested to exert a stretching force on the substrate, which remains bound to several apical domains during their motion (44). This stretching action is thought to forcefully unfold GroEL substrates, rescuing kinetically trapped folding intermediates (6, 16). Our previous study of GroEL-ATP suggested a possible mechanism for forced unfolding in which the radial expansion of subunits exposed bound substrate to stretching (8). Our structure of GroEL-ADP·BeF_3_-Rubisco suggests a different geometrical pathway of stretching. A substrate that is multivalently bound between GroEL apical domains and C termini could be stretched during their transition from Rs1 to GroES-bound states. In support of this, it has previously been shown that forced unfolding of Rubisco is attenuated when the C termini are removed (16). Bound Rubisco might also be destabilised due to the exclusion of bulk water from the occupied GroEL cavity, reducing the hydrophobic effect and altering the energetics of its folding relative to that in bulk solution (45). Following GroES binding, non-native Rubisco would be released into the folding chamber where it may still associate with the C termini (19).

Our reconstructions of GroEL-ADP·AlF_3_-Rubisco-GroES showed Rubisco in the upper half of the folding chamber, held in place by interactions with charged and hydrophobic residues located in the GroEL apical domains (K226, E255, F281, and Y360), and in GroES (Y71). Due to the low local resolution of the encapsulated Rubisco, we could only reliably identify a native-like conformation in a small subset of particles. Several cryoEM structures of GroEL-GroES-substrate complexes, including two with Rubisco as the substrate protein, have been published (10, 15, 19). Importantly, the published structure of GroEL_43Py398A_-GroES (15) is not a fully folding-active complex and instead represents a stalled complex immediately prior to the release of the substrate inside the folding chamber. Both previously published structures of GroEL-ADP·AlF_3_-Rubisco-GroES showed either non-native or native-like Rubisco located in the lower half of the folding chamber, interacting with residues of the GroEL apical domains (F281, Y360), the equatorial domains (F44), and the GroEL C termini (15, 19). GroEL residue F281 appears to be critical in the folding of substrate proteins. This is supported by earlier work showing that the mutant GroEL_F281D_ supports binding of non-native substrate, but exhibits decreased ATPase activity, reduced folding, and aggregation of the substrate protein upon its release (4).

The position of Rubisco in GroEL-GroES was similar to that of the T4 bacteriophage capsid protein, gp23 (10), also observed in a native-like state. Rubisco interacted with several of the GroES Y71 residues that together form a hydrophobic ring on the folding chamber ceiling. Previous work has shown that this hydrophobic ring may take part in the folding process for some GroEL substrates (32). We did not observe contacts with hydrophobic residues in the lower part of the chamber, such as F44 or the C termini, and instead observed interactions with charged residues in the GroEL apical domains. Encapsulated substrates may start as folding intermediates at the bottom of the GroEL-GroES cavity, sequestered primarily by the C termini and the F44 loop. As folding proceeds and hydrophobic residues in the substrate become buried, the interaction with the C termini might diminish, allowing the substrate to occupy a more central or upper position in the cavity. The Rubisco intermediate in our class II structure might represent a near-native state, with some distortion of the domain interface, primed for release following detachment of GroES.

## Conclusion

Our results, benefitting from the substantial advances in cryoEM methodology in the last decade, provide a more detailed view of the chaperonin-assisted folding pathway and mechanism for a major, model substrate. Our cryoEM reconstructions show the progression through key initial steps in the nucleotide cycle and the changing sites of substrate interaction within the complex through the folding reaction, as well as substrate displacement in the GroEL-GroES cavity as native structure is formed.

## Methods

### Protein Expression and Purification.

Expression and purification of *E. coli* GroEL and GroES and *R. rubrum* Rubisco are described in *SI Appendix*, *Supplementary Materials and Methods*.

### Formation of GroEL-Rubisco Binary Complexes.

Rubisco was unfolded in unfolding buffer (50 mM HEPES-KOH, pH 7.5, 8 M urea) at 21 °C for at least 30 min. Binary complexes of GroEL bound to non-native Rubisco were prepared by diluting non-native Rubisco into chloride-free GroEL-containing HKM buffer (50 mM HEPES-KOH, pH 7.5, 10 mM KOAc, 10 mM Mg(OAc)2, 2 mM DTT + 1 µM GroEL tetradecamer). Unfolded Rubisco was added to 1 mL of GroEL-containing HKM buffer in five 2 µL additions. Gentle mixing and centrifugation were performed after each addition. After the fifth addition, the final concentration of Rubisco was 4 µM, a fourfold molar excess over GroEL. The sample was incubated at 21 °C for 10 min with periodic mixing via gentle pipetting. Complexes were centrifuged at 16,200 RCF at 21 °C for 10 min to pellet aggregated protein. The presence of binary complexes was confirmed by native PAGE and native mass spectrometry. Binary complexes were freshly prepared for all cryoEM experiments.

### Mass Spectrometry.

Samples for native mass spectrometry were exchanged into 50 mM ammonium acetate (pH 6.8) using 10-kDa cutoff Amicon Ultra centrifugal filtration units (Merck Millipore). Samples were introduced to a first-generation Waters Synapt QToF (Waters Corporation, UK) in nano electrospray gold-coated borosilicate glass capillaries (prepared in-house). Mass calibration was performed using a solution of 30 mg/mL caesium iodide (Fluka). Typical machine parameters used were capillary 1.4 kV, sampling cone 150 V, extraction cone 4.5 V, backing pressure 7.5 mbar, trap CE 40 eV, transfer CE 10 eV, bias 88 V, source wave velocity 300 ms^−1^, source wave height 0.2 V, trap wave velocity 300 ms^−1^, and trap wave height 0.2 V. Spectra were analysed using MassLynx v4.1 (Waters Corporation, UK) and Amphitrite (46). Spectra were loaded into Amphitrite using a grain size of 3 and a smoothing value of 2.

### CryoEM Sample Preparation, Data Collection and Analysis.

#### GroEL-Rubisco.

GroEL-Rubisco was prepared and concentrated to 3.4 μM. Grids were prepared using a Chameleon instrument (SPT Labtech). We collected data from two grids frozen at different dispense-to-freeze times. Grid 1 was frozen at 1,039 ms and grid 2 was frozen at 101 ms. Movies (48 frames) were collected using the EPU software on a Titan Krios transmission electron microscope (Thermo Fisher Scientific) operating at 300 keV, equipped with a Gatan K2 Summit direct electron detector in counting mode and Gatan energy filter. The defocus range was set between −1.4 and −3.0 μm, and the total exposure was 40.2 electrons/Å^2^. Images were recorded at a pixel size of 1.34 Å/pixel.

#### GroEL-ATP-Rubisco.

UltrAuFoil R2/2 grids were glow discharged at 30 mA for 60 s using a Pelco easiGlow (Ted Pella, Inc., USA) system. ATP (3 mM) was added to GroEL-Rubisco (1 μM). Three microlitres of the mixture was applied to grids, blotted, and plunged into liquid ethane cooled by liquid nitrogen using a Vitrobot mark IV (Thermo Fisher Scientific, USA) operating at 100% humidity and 4 °C. Blot time was set to 5 s, blot force set to −10. The time between adding ATP to GroEL-Rubisco and plunge-freezing was approximately 10 s. Movies (50 frames) were collected using the EPU software on a Titan Krios transmission electron microscope (Thermo Fisher Scientific) operating at 300 keV, equipped with a Gatan K3 direct electron detector operating in super-resolution mode and a Gatan energy filter. The defocus range was set between −1.5 and −2.7 μm, and the total exposure was 50 electrons/Å^2^. Images were recorded at a pixel size of 1.06 Å/pixel. A stage tilt of 35° was set at the start of image acquisition.

#### GroEL-ADP·BeF_3_-Rubisco.

GroEL-Rubisco complexes were prepared and concentrated to 7 μM. We then added 3 mM ADP, 20 mM KF, and 2 mM BeSO_4_ and incubated the sample for 10 min. Grids were prepared using a Chameleon instrument (SPT Labtech) with a dispense-to-freeze time of 54 ms. Movies (50 frames) were collected using the EPU software on a Titan Krios transmission electron microscope (Thermo Fisher Scientific) operating at 300 keV, equipped with a Gatan K3 direct electron detector operated in super-resolution mode and a Gatan energy filter. The defocus range was set between −1.5 and −2.7 μm, and the total exposure was 50 electrons/Å^2^. Images were recorded at a pixel size of 1.068 Å/pixel.

#### GroEL-ADP·AlF_3_-Rubisco-GroES-ADP·AlF_3_.

GroEL-Rubisco complexes were prepared and concentrated to 7 μM. We then added 7 μM GroES, 3 mM ADP, 20 mM KF, and 2 mM KAl(SO_4_)_2_ and incubated the sample for 10 min. Grids were prepared using a Chameleon instrument (SPT Labtech) with a dispense-to-freeze time of 54 ms. Movies (50 frames) were collected using the EPU software on a Titan Krios transmission electron microscope (Thermo Fisher Scientific) operating at 300 keV, equipped with a Gatan K3 direct electron detector operated in super-resolution mode and a Gatan energy filter. The defocus range was set between −1.5 and −2.7 μm, and the total exposure was 72 electrons/Å^2^. Images were recorded at a pixel size of 0.828 Å/pixel.

#### CryoEM image processing.

The initial approach for image processing was the same for all datasets. Full image processing details for individual datasets are described in *SI Appendix*, *Supplementary Materials and Methods*. Micrograph movies were corrected for beam-induced motion using Motioncorr2 (46). For movies collected in super-resolution mode using a Gatan K3 camera, micrographs were downsampled by a factor of 2 during motion correction. The CTF parameters of motion-corrected micrographs were estimated using Gctf (47). Particles were picked using the neural network particle picker included in EMAN v.2.2 (48). Particle coordinates (.box files) were imported into RELION v.3.1 (49). Particles were typically extracted from micrographs with 2 to 3 times downsampling, giving pixel sizes of 2 to 4 Å/pixel. We used downsampled particles for initial 2D classification, then re-extracted particles with finer sampling for 3D classification and final 3D refinements. Downsampled particles were imported into cryoSPARC (50) and subjected to three rounds of reference-free 2D classification. Particles from featureless, noisy, or poorly resolved classes were discarded. Good particles from 2D classification were imported back into Relion using the csparc2star.py Python script (51). Subsequent image processing steps were performed in Relion v.3.1 or cryoSPARC v.3.3.1. No symmetry was applied during any step of image processing. For 3D refinements, an initial model of GroEL or GroEL-GroES was generated from a previously published cryoEM reconstruction (EMDB: 3415 and EMDB: 2325), or generated using ab initio reconstruction in cryoSPARC, and low-pass filtered to 30 to 60 Å.

#### Model building.

Model building details are provided in *SI Appendix*, *Supplementary Materials and Methods*.

## Supplementary Material

Appendix 01 (PDF)

## Data Availability

The cryo-EM maps and associated coordinates have been deposited in the EMDB and on the PDB: GroEL-Rubisco (EMDB: 15939, PDB: 8BA7), GroEL-ADP·BeF_3_-Rubisco (EMDB: 15940, PDB: 8BA8), GroEL-ATP-Rubisco (EMDB: 15941), GroEL-ADP·AlF_3_-Rubisco-GroES (EMDB: 15942, PDB: 8BA9), GroEL-ADP·AlF_3_-Rubisco-GroES class I (EMDB: 15943), GroEL-ADP·AlF_3_-Rubisco-GroES class II (EMDB: 15944, PDB: 8BAA), GroEL-ADP·AlF_3_-Rubisco-GroES class III (EMDB: 15945), and GroEL-ADP·AlF_3_-Rubisco-GroES class IV (EMDB: 15945).

## References

[1] H. Saibil, Chaperone machines for protein folding, unfolding and disaggregation. Nat. Rev. Mol. Cell Biol. **14**, 630–642 (2013). 24026055 10.1038/nrm3658 PMC4340576

[2] A. L. Horwich, W. A. Fenton, Chaperonin-assisted protein folding: A chronologue. Q. Rev. Biophys. **53**, e4 (2020). 32070442 10.1017/S0033583519000143

[3] K. Braig , The crystal structure of the bacterial chaperonln GroEL at 2.8 Å. Nature **371**, 578–586 (1994). 7935790 10.1038/371578a0

[4] W. A. Fenton, Y. Kashi, K. Furtak, A. L. Norwich, Residues in chaperonin GroEL required for polypeptide binding and release. Nature **371**, 614–619 (1994). 7935796 10.1038/371614a0

[5] J. F. Hunt, A. J. Weaver, S. J. Landry, L. Gierasch, J. Deisenhofer, The crystal structure of the GroES co-chaperonin at 2.8 Å resolution. Nature **379**, 37–45 (1996). 8538739 10.1038/379037a0

[6] Z. Lin, D. Madan, H. S. Rye, GroEL stimulates protein folding through forced unfolding. Nat. Struct. Mol. Biol. **15**, 303–311 (2008). 18311152 10.1038/nsmb.1394 PMC3744391

[7] D. Thirumalai, G. H. Lorimer, C. Hyeon, Iterative annealing mechanism explains the functions of the GroEL and RNA chaperones. Protein Sci. **29**, 360–377 (2020). 31800116 10.1002/pro.3795 PMC6954723

[8] D. K. Clare , ATP-triggered conformational changes delineate substrate-binding and -folding mechanics of the GroEL chaperonin. Cell **149**, 113–123 (2012). 22445172 10.1016/j.cell.2012.02.047 PMC3326522

[9] N. Elad , Topologies of a substrate protein bound to the chaperonin GroEL. Mol. Cell **26**, 415–426 (2007). 17499047 10.1016/j.molcel.2007.04.004 PMC1885994

[10] D. K. Clare, P. J. Bakkes, H. van Heerikhuizen, S. M. van der Vies, H. R. Saibil, Chaperonin complex with a newly folded protein encapsulated in the folding chamber. Nature **457**, 107–110 (2009). 19122642 10.1038/nature07479 PMC2728927

[11] J. Weaver , GroEL actively stimulates folding of the endogenous substrate protein PepQ. Nat. Commun. **8**, 15934 (2017). 28665408 10.1038/ncomms15934 PMC5497066

[12] R. Natesh, D. K. Clare, G. W. Farr, A. L. Horwich, H. R. Saibil, A two-domain folding intermediate of RuBisCO in complex with the GroEL chaperonin. Int. J. Biol. Macromol. **118**, 671–675 (2018). 29959019 10.1016/j.ijbiomac.2018.06.120 PMC6096091

[13] D. Balchin, G. Miličić, M. Strauss, M. Hayer-Hartl, F. U. Hartl, Pathway of actin folding directed by the eukaryotic chaperonin TRiC. Cell **174**, 1507–1521.e16 (2018). 30100183 10.1016/j.cell.2018.07.006

[14] A. A. Mamchur , Structural and computational study of the GroEL–Prion protein complex. Biomedicines **9**, 1649 (2021). 34829878 10.3390/biomedicines9111649 PMC8615626

[15] D.-H. Chen , Visualizing GroEL/ES in the act of encapsulating a folding protein. Cell **153**, 1354–1365 (2013). 23746846 10.1016/j.cell.2013.04.052 PMC3695626

[16] J. Weaver, H. S. Rye, The C-terminal tails of the bacterial chaperonin GroEL stimulate protein folding by directly altering the conformation of a substrate protein. J. Biol. Chem. **289**, 23219–23232 (2014). 24970895 10.1074/jbc.M114.577205 PMC4132819

[17] Z. Xu, A. L. Horwich, P. B. Sigler, The crystal structure of the asymmetric GroEL–GroES–(ADP)7 chaperonin complex. Nature **388**, 741–750 (1997). 9285585 10.1038/41944

[18] M. J. Kerner , Proteome-wide analysis of chaperonin-dependent protein folding in Escherichia coli. Cell **122**, 209–220 (2005). 16051146 10.1016/j.cell.2005.05.028

[19] H. Kim , Cryo-EM structures of GroEL:ES2 with RuBisCO visualize molecular contacts of encapsulated substrates in a double-cage chaperonin. iScience **25**, 103704 (2022). 35036883 10.1016/j.isci.2021.103704 PMC8749442

[20] S. M. van der Vies, P. V. Viitanen, A. A. Gatenby, G. H. Lorimer, R. Jaenicke, Conformational states of ribulosebisphosphate carboxylase and their interaction with chaperonin 60. Biochemistry **31**, 3635–3644 (1992). 1348956 10.1021/bi00129a012

[21] V. P. Dandey , Spotiton: New features and applications. J. Struct. Biol. **202**, 161–169 (2018). 29366716 10.1016/j.jsb.2018.01.002 PMC6317895

[22] D. P. Klebl , Need for speed: Examining protein behavior during CryoEM grid preparation at different timescales. Structure **28**, 1238–1248.e4 (2020). 32814033 10.1016/j.str.2020.07.018 PMC7652391

[23] I. Kass, A. Horovitz, Mapping pathways of allosteric communication in GroEL by analysis of correlated mutations. Proteins **48**, 611–617 (2002). 12211028 10.1002/prot.10180

[24] J. Wang, L. Chen, Domain motions in GroEL upon binding of an oligopeptide. J. Mol. Biol. **334**, 489–499 (2003). 14623189 10.1016/j.jmb.2003.09.074

[25] Y. Z. Tan , Addressing preferred specimen orientation in single-particle cryo-EM through tilting. Nat. Methods **14**, 793–796 (2017). 28671674 10.1038/nmeth.4347 PMC5533649

[26] C. Chaudhry , Role of the γ-phosphate of ATP in triggering protein folding by GroEL–GroES: Function, structure and energetics. EMBO J. **22**, 4877–4887 (2003). 14517228 10.1093/emboj/cdg477 PMC204461

[27] H. Taguchi, K. Tsukuda, F. Motojima, A. Koike-Takeshita, M. Yoshida, BeFx stops the chaperonin cycle of GroEL-GroES and generates a complex with double folding chambers. J. Biol. Chem. **279**, 45737–45743 (2004). 15347650 10.1074/jbc.M406795200

[28] Y. Gomez-Llorente , Structural basis for active single and double ring complexes in human mitochondrial Hsp60-Hsp10 chaperonin. Nat. Commun. **11**, 1916 (2020). 32317635 10.1038/s41467-020-15698-8 PMC7174398

[29] R. Sanchez-Garcia , DeepEMhancer: A deep learning solution for cryo-EM volume post-processing. Commun. Biol. **4**, 1–8 (2021). 34267316 10.1038/s42003-021-02399-1 PMC8282847

[30] P. D. Kiser, G. H. Lorimer, K. Palczewski, Use of thallium to identify monovalent cation binding sites in GroEL. Acta Crystallogr. Sect. F Struct. Biol. Cryst. Commun. **65**, 967–971 (2009). 10.1107/S1744309109032928 PMC2765879 19851000

[31] L. Ditzel , Crystal structure of the thermosome, the archaeal chaperonin and homolog of CCT. Cell **93**, 125–138 (1998). 9546398 10.1016/s0092-8674(00)81152-6

[32] J. D. Wang, C. Herman, K. A. Tipton, C. A. Gross, J. S. Weissman, Directed evolution of substrate-optimized GroEL/S chaperonins. Cell **111**, 1027–1039 (2002). 12507429 10.1016/s0092-8674(02)01198-4

[33] S. S. Kudryavtseva , Novel cryo-EM structure of an ADP-bound GroEL–GroES complex. Sci. Rep. **11**, 18241 (2021). 34521893 10.1038/s41598-021-97657-x PMC8440773

[34] E. Mizohata , Crystal structure of activated ribulose-1,5-bisphosphate carboxylase/oxygenase from green alga Chlamydomonas reinhardtii complexed with 2-carboxyarabinitol-1,5-bisphosphate. J. Mol. Biol. **316**, 679–691 (2002). 11866526 10.1006/jmbi.2001.5381

[35] E. van Duijn , Tandem mass spectrometry of intact GroEL−substrate complexes reveals substrate-specific conformational changes in the trans ring. J. Am. Chem. Soc. **128**, 4694–4702 (2006). 16594706 10.1021/ja056756l

[36] E. van Duijn, A. J. R. Heck, S. M. van der Vies, Inter-ring communication allows the GroEL chaperonin complex to distinguish between different substrates. Protein Sci. **16**, 956–965 (2007). 17456746 10.1110/ps.062713607 PMC2206630

[37] A. M. Roseman, S. Chen, H. White, K. Braig, H. R. Saibil, The chaperonin ATPase cycle: Mechanism of allosteric switching and movements of substrate-binding domains in GroEL. Cell **87**, 241–251 (1996). 8861908 10.1016/s0092-8674(00)81342-2

[38] D. C. Boisvert, J. Wang, Z. Otwinowski, A. L. Horwich, P. B. Sigler, The 2.4 A crystal structure of the bacterial chaperonin GroEL complexed with ATP gamma S. Nat. Struct. Biol. **3**, 170–177 (1996). 8564544 10.1038/nsb0296-170

[39] H. S. Rye , Distinct actions of cis and trans ATP within the double ring of the chaperonin GroEL. Nature **388**, 792–798 (1997). 9285593 10.1038/42047

[40] M. Jin , An ensemble of cryo-EM structures of TRiC reveal its conformational landscape and subunit specificity. Proc. Natl. Acad. Sci. U.S.A. **116**, 19513–19522 (2019). 31492816 10.1073/pnas.1903976116 PMC6765261

[41] C. Chaudhry, A. L. Horwich, A. T. Brunger, P. D. Adams, Exploring the structural dynamics of the E.coli chaperonin GroEL using translation-libration-screw crystallographic refinement of intermediate states. J. Mol. Biol. **342**, 229–245 (2004). 15313620 10.1016/j.jmb.2004.07.015

[42] B. P. Burnett, A. L. Horwich, K. B. Low, A carboxy-terminal deletion impairs the assembly of GroEL and confers a pleiotropic phenotype in Escherichia coli K-12. J. Bacteriol. **176**, 6980–6985 (1994). 7961461 10.1128/jb.176.22.6980-6985.1994 PMC197070

[43] X. Fei, D. Yang, N. LaRonde-LeBlanc, G. H. Lorimer, Crystal structure of a GroEL-ADP complex in the relaxed allosteric state at 2.7 Å resolution. Proc. Natl. Acad. Sci. U.S.A. **110**, E2958–E2966 (2013). 23861496 10.1073/pnas.1311996110 PMC3740897

[44] F. Motojima, C. Chaudhry, W. A. Fenton, G. W. Farr, A. L. Horwich, Substrate polypeptide presents a load on the apical domains of the chaperonin GroEL. Proc. Natl. Acad. Sci. U.S.A. **101**, 15005–15012 (2004). 15479763 10.1073/pnas.0406132101 PMC523455

[45] I. Korobko, R. B. Eberle, M. Roy, A. Horovitz, A diminished hydrophobic effect inside the GroEL/ES cavity contributes to protein substrate destabilization. Proc. Natl. Acad. Sci. U.S.A. **119**, e2213170119 (2022). 36409898 10.1073/pnas.2213170119 PMC9860310

[46] G. N. Sivalingam, J. Yan, H. Sahota, K. Thalassinos, Amphitrite: A program for processing travelling wave ion mobility mass spectrometry data. Int. J. Mass Spectrom. **345–347**, 54–62 (2013). 10.1016/j.ijms.2012.09.005 PMC4375678 25844045

[47] S. Q. Zheng , MotionCor2–Anisotropic correction of beam-induced motion for improved cryo-electron microscopy. Nat. Methods **14**, 331–332 (2017). 28250466 10.1038/nmeth.4193 PMC5494038

[48] K. Zhang, Gctf: Real-time CTF determination and correction. J. Struct. Biol. **193**, 1–12 (2016). 26592709 10.1016/j.jsb.2015.11.003 PMC4711343

[49] J. M. Bell, M. Chen, T. Durmaz, A. C. Fluty, S. J. Ludtke, New software tools in EMAN2 inspired by EMDatabank map challenge. J. Struct. Biol. **204**, 283–290 (2018). 30189321 10.1016/j.jsb.2018.09.002 PMC6163079

[50] J. Zivanov , New tools for automated high-resolution cryo-EM structure determination in RELION-3. Elife **7**, e42166 (2018). 30412051 10.7554/eLife.42166 PMC6250425

[51] A. Punjani, J. L. Rubinstein, D. J. Fleet, M. A. Brubaker, cryoSPARC: Algorithms for rapid unsupervised cryo-EM structure determination. Nat. Methods **14**, 290–296 (2017). 28165473 10.1038/nmeth.4169

