# Supplementary material for: Structural basis of substrate progression through the bacterial chaperonin cycle
Source: Proc Natl Acad Sci U S A. 2023 Dec 8;120(50):e2308933120. doi: 10.1073/pnas.2308933120 (PMC10723157; doi:10.1073/pnas.2308933120)
Supplement: Supplementary file 1 — Appendix 01 (PDF) [file pnas.2308933120.sapp.pdf]

SUPPLEMENTARY MATERIALS AND METHODS

Protein expression and purification

Escherichia coli GroEL

Plasmid pTrcESL, a kind gift from Dr. Peter Lund (University of Birmingham, UK), was transformed into BL21 E. coli. Cells were grown in LB media at 37 °C using baffled flasks. Overexpression of GroELS was induced by addition of 0.5 mM isopropyl β-D-thiogalactopyranoside (IPTG) at an A600 of 0.6 - 0.8. After 3.5 hours cells were harvested by centrifugation, resuspended in chilled lysis buffer (50 mM Tris, pH 7.4), and disrupted using an EmulsiFlex-C3 (Avestin, Ottawa, Canada). Cellular debris and insoluble material were removed by centrifugation at 55,000 RCF at 4 °C for 1 hour. Subsequent purification and dialysis steps were performed at 4 °C unless stated. Soluble lysate was filtered through a 0.45 μm syringe filter and loaded onto a 140 mL Q Sepharose FastFlow ion-exchange column (GE Healthcare) equilibrated in buffer A (50 mM Tris, pH 7.4, 1 mM DTT). The column was washed with 20% buffer B (50 mM Tris, pH 7.4, 1 mM DTT, 1M NaCl) until A280 reached a steady baseline. Protein was eluted with a 20 - 50% 15 column volume gradient of buffer B and fractions were collected. Elution of GroEL was confirmed by SDS-PAGE and fractions corresponding to the latter half of the GroEL peak were pooled. Solid ammonium sulphate was added slowly to the pooled fractions to a final concentration of 1.2 M and stirred overnight. Pooled fractions from ion-exchange chromatography were filtered through a 5 μm syringe filter and loaded onto a 22 mL Source 15ISO hydrophobic interaction column (GE Healthcare) equilibrated in buffer C (50 mM Tris, pH 7.4, 1 mM DTT, 1.2 M NH4(SO4)2. The column was washed with buffer C until the A280 reached a steady baseline < 50 mAU. GroEL was eluted with a 14 column volume reverse-gradient of 100 - 0% buffer C to buffer A. Peak fractions were analysed by SDS-PAGE. The protein concentration and tryptophan fluorescence of each peak fraction were measured. Since GroEL has no tryptophan residues, tryptophan fluorescence is an indicator of contamination by bound Trp-containing proteins. Tryptophan fluorescence was measured using a FluoroMax 3 (Horiba). Protein concentration was measured by Pierce BSA protein assay. Fractions with high protein concentration and low fluorescence were pooled and dialysed overnight against 4 L of buffer D (50 mM Tris, pH 7.4, 50 mM KCl, 1 mM DTT). The final peak fractions from hydrophobic interaction chromatography typically yielded the purest GroEL. To further strip GroEL-bound contaminating proteins, the dialysed protein was concentrated to 20 mg/mL, then 2 mL of buffer E (1:1 mixture of buffer D and Affi-gel Blue resin) was added per 30 mg of total protein. Methanol was then added dropwise to 20% v/v while stirring. After mixing, the sample was rocked gently at room temperature (~ 21 °C) for 2 hours. GroEL was recovered in the supernatant by filtration through a 1.2 μm syringe filter and the resin was washed once with its volume of buffer D. Eluates were pooled and dialysed overnight against 4 L of buffer D. Purified GroEL was filter-sterilised and concentrated to 30 - 40 mg/mL. Aliquots of concentrated GroEL were flash frozen in liquid nitrogen and stored at -80 °C until use.

Escherichia coli GroES

Plasmid pTrcESL was transformed into BL21 E. coli. Cells were grown in LB media at 37 °C using baffled flasks. Overexpression of GroELS was induced by addition of 0.5 mM IPTG at an A600 of 0.6 - 0.8. After 3.5 hours cells were harvested by centrifugation, resuspended in chilled lysis buffer (50 mM Tris, pH 7.4), and disrupted using an EmulsiFlex-C3 (Avestin, Ottawa, Canada). Cellular debris and insoluble material were removed by centrifugation at 55,000 RCF at 4 °C for 1 hour. Soluble lysate was transferred to a fresh tube and acidified to pH 5.0 by dropwise addition of 1 M acetic acid. Precipitated material was removed by centrifugation at 25,000 RCF at 4 °C for 15 min. Subsequent purification and dialysis steps were performed at 4 °C unless stated. Acidified soluble lysate was filtered through a 0.45 μm syringe filter and loaded onto a 125 mL SP Sepharose FastFlow ion-exchange column (GE Healthcare) equilibrated in buffer A (50 mM NaOAc, pH 5.0). The column was washed with buffer A until A280 reached a steady baseline. Protein was eluted with a 0 - 50% 12.5 column volume gradient of buffer B (50 mM NaOAc, pH 5.0, 1 M NaCl) and fractions were collected. Elution of GroES was confirmed by SDS-PAGE. Fractions that corresponded to the GroES peak were pooled, neutralised to pH 7.5 - 8.0 with Tris-base, and dialysed overnight against 4 L of buffer C (50 mM Tris, pH 7.4). Dialysed protein was loaded onto a 24 mL Source 15Q ion-exchange column equilibrated in buffer C. The column was washed with buffer C until the A280 reached a steady baseline < 50 mAU. GroES was eluted with a 0 - 50% 20 CV gradient of buffer D (50 mM Tris, pH 7.4, 1 M NaCl). Elution of GroES was confirmed by SDS-PAGE. Fractions that corresponded to GroES were pooled and dialysed overnight against 4 L buffer C. Purified GroES was filter-sterilised and concentrated to 15 mg/mL. Aliquots of concentrated GroES were flash frozen in liquid nitrogen and stored at -80 °C until use.

Rhodospirillum rubrum RuBisCO

Plasmid pT7Rub, a kind gift from Dr. Wayne Fenton (Yale University, USA), was transformed into BL21 (DE3) E. coli. Cells were grown in LB media in baffled flasks at 20 °C for 24 hours in the absence of induction. Cells were harvested by centrifugation, resuspended in lysis buffer (50 mM Tris, pH 7.4), and disrupted using an EmulsiFlex-C3 (Avestin, Ottawa, Canada). Cellular debris and insoluble components were removed by centrifugation at 55,000 RCF at 4 °C for 1 hour. Subsequent purification and dialysis steps were performed at 4 °C unless stated. Soluble lysate was filtered through a 0.45 μm syringe filter and loaded onto a 140 mL Q Sepharose FastFlow ion-exchange column (GE Healthcare) equilibrated in buffer A (50 mM Tris, pH 7.4, 1 mM DTT). The column was washed with buffer A (50 mM Tris, pH 7.4, 1 mM DTT) until A280 reached a steady baseline. Rubisco was eluted with a 0 - 50% 10 column volume gradient of buffer B (50 mM Tris, pH 7.4, 1 mM DTT, 1M NaCl) and fractions were collected. Elution of Rubisco was confirmed by SDS-PAGE and peak fractions were pooled and dialysed overnight against 4L of buffer C (20 mM Tris, pH 7.4, 1 mM DTT). Pooled fractions from ion-exchange chromatography were filtered through a 1.2 μm syringe filter and loaded onto a 60 mL Affi-Gel Blue column equilibrated in buffer C. The column was washed with buffer C until

the A280 reached a steady baseline. Rubisco was eluted with a 0 - 50% 4 column volume gradient of buffer D (20 mM Tris, pH 7.4, 1 mM DTT, 1M NaCl). Peak fractions were analysed by SDS-PAGE, pooled, and dialysed overnight against 4 L of buffer A. Pooled fractions from dye-ligand chromatography were filtered through a 1.2 µm syringe filter and loaded onto a 24 mL Source 15Q column equilibrated in buffer A. The column was washed with buffer A until the A280 reached a steady baseline. Rubisco was eluted with a 0 - 50% 10 column volume gradient of buffer B. Peak fractions were analysed by SDS-PAGE and the purest fractions were pooled and dialysed overnight against 4 L of buffer A. Purified Rubisco was concentrated to 10 - 20 mg/mL and filter-sterilised. Aliquots of concentrated Rubisco were flash frozen in liquid nitrogen and stored at -80 °C until use.

CryoEM image processing

The same general approach for image processing was used for all data sets. Micrograph movies were corrected for beam induced motion using Motioncorr2 (1). For movies collected in super-resolution mode using a Gatan K3 camera, micrographs were down sampled by a factor of 2 during motion correction. The CTF parameters of motion-corrected micrographs were estimated using Gctf (2). Particles were picked using the neural network particle picker included in EMAN v.2.2 (3). Particle coordinates (.box files) were imported into RELION v.3.1 (4). Particles were typically extracted from micrographs with 2 - 3 times down-sampling, giving pixel sizes of 2 - 4 Å/pixel. We used down-sampled particles for initial 2D classification, then re-extracted particles at smaller pixel sizes for 3D classification and final 3D refinements. Down-sampled particles were imported into cryoSPARC (5) and subjected to three rounds of reference-free 2D classification. Particles from featureless, noisy, or poorly resolved classes were discarded. Good particles from 2D classification were imported back into Relion using the csparc2star.py Python script (6). Subsequent image processing steps were performed in Relion v.3.1 or cryoSPARC v.3.3.1. No symmetry was applied during any step of image processing. For 3D refinements, an initial model of GroEL or GroEL-GroES was generated from a previously published cryoEM reconstruction (EMDB: 3415 and EMDB: 2325) and low-pass filtered to 30 - 60 Å.

GroEL-Rubisco

The image processing workflow for GroEL-Rubisco is summarised in **Fig. S4**. For GroEL-Rubisco, two data sets from different Chameleon grids were collected. We processed each data set separately and combined particles following 3D classification. For grid 1 (dispense-to-freeze time = 1039 ms), 175,121 particles were extracted from motion-corrected micrographs at a pixel size of 2.68 Å/pixel (384x384 pixel box, rescaled to 192x192 pixels) using Relion. Extracted particles were subjected to several rounds of iterative 2D classification in cryoSPARC. Following 2D classification, 137,331 particles were imported back into Relion, re-extracted at 1.34 Å/pixel (256x256 pixel box), and refined to 5.5 Å. The particles were then subjected to CTF refinement and refined to 4.6 Å. Particles were subjected to 3D classification using 4 classes and a 200 Å circular mask. One class (51,241 particles) displayed strong density for non-native Rubisco in the GroEL

cavity and was selected. For grid 2 (dispense-to-freeze time = 101 ms), 233,763 particles were extracted from motion-corrected micrographs at a pixel size of 2.68 Å/pixel (384x384 pixel box, rescaled to 192x192 pixels) using Relion. Extracted particles were subjected to iterative 2D classification in cryoSPARC. Following 2D classification, 67,702 particles were imported back into Relion, re-extracted at 1.34 Å/pixel (256x256 pixel box), and refined to 7.3 Å. Particles were then subjected to CTF refinement, and refined to 7.0 Å. Particles were subjected to 3D classification using 4 classes and a 200 Å circular mask. One class (14,212 particles) displayed strong density for non-native Rubisco in the GroEL cavity and was selected. The selected 3D classes from the grid 1 and grid 2 data sets were combined (65,453 particles), re-extracted at 1.34 Å/pixel in a larger box (384x384 pixels), and refined to 4.4 Å. Local resolution estimation and local filtering was performed using Relion.

GroEL-ATP-Rubisco

The image processing workflow for GroEL-ATP-Rubisco is summarised in **Fig. S5**. For GroEL-ATP-Rubisco, a total of 2,267,111 particles were extracted from motion-corrected micrographs at a pixel size of 2.12 Å/pixel (256x256 pixel box, rescaled to 128x128 pixels) using Relion. Extracted particles were subjected to iterative 2D classification using cryoSPARC. Following 2D classification, 961,840 particles were imported back into Relion and refined to a resolution of 4.2 Å. In the consensus refinement, the apical domains of GroEL rings were poorly resolved. We attributed this to partial denaturation of GroEL-ATP at the air-water interface. Particles were subjected to a first round of 3D classification using eight classes. Six classes showed a poorly resolved GroEL complex resembling the consensus reconstruction. We identified two classes in which both GroEL rings were comparatively well resolved. Each of these classes displayed inter-ring asymmetry, with both a narrow ring and a wide ring, reminiscent of our previous work on GroEL-ATP (7). In class IV (105,302 particles) the wide ring was best resolved, and in class V (97,460 particles) the narrow ring was best resolved. Particles from class IV were refined to 5.3 Å, however the narrow ring was still poorly resolved. We aimed to identify subsets of particles in class IV in which the narrow ring of GroEL was intact. To do this we subtracted the better resolved wide ring from class IV particles, then performed a second round of 3D classification (skipping alignments) using eight classes. In the second round of 3D classification, classes III, IV, and VIII showed stronger narrow ring density. Particles from these 3D classes were reverted to their original non-subtracted images and refined. At a resolution of 6.5 Å, class III (13,015 particles) was the best resolved of these and displayed the first hints of intra-ring asymmetry. Class III particles were re-extracted at 1.7 Å/pixel (256x256 pixels rescaled to 160x160), imported into CryoSPARC and refined to 4.3 Å. The local resolution was estimated using tools in CryoSPARC and a locally filtered map was calculated. We experimented with combining different classes of particles, and with using the same particle subtraction strategy on class V from the first round of 3D classification, but these efforts led to decreased resolution.

GroEL-ADP·BeF$_3$-Rubisco

The image processing workflow for GroEL-ADP·BeF_3-Rubisco is summarised in **Fig. S6**. For GroEL-ADP·BeF_3-Rubisco, two data sets were collected from different Chameleon grids, both with a dispense-to-freeze time of 54 ms. We processed each data set separately and combined particles following the second round of 3D classification. For grid 1, a total of 3,616,365 particles were extracted from motion-corrected micrographs at a pixel size of 3.2 Å/pixel (384x384 pixel box, rescaled to 128x128 pixels) using Relion. Extracted particles were subjected to iterative 2D classification in cryoSPARC. Following 2D classification, 2,588,967 particles were imported back into Relion and refined to 6.4 Å. Particles were subjected to 3D classification using 4 classes and a 220 Å circular mask. One class (677,383 particles) showed a well resolved GroEL complex. Particles from this class were re-extracted at 2.0 Å/pixel (468x468 pixel box, rescaled to 256x256 pixels) using Relion and refined to 4.5 Å, then subjected to CTF refinement and refined to 4.0 Å. A second round of 3D classification using a soft solvent mask was used to identify the best resolved particles in data set 1. Two classes (350,876 particles) showed a well-resolved GroEL complex and were selected for further processing. For grid 2, a total of 4,096,257 particles were extracted from motion-corrected micrographs at a pixel size of 3.2 Å/pixel (384x384 pixel box, rescaled to 128x128 pixels) using Relion. Extracted particles were subjected to iterative 2D classification in cryoSPARC. Following 2D classification, 1,792,697 particles were imported back into Relion and refined to 6.5 Å. Particles were subjected to 3D classification using 4 classes and a 220 Å circular mask. One class (592,763 particles) showed a well resolved GroEL complex. Particles from this class were re-extracted at 2.0 Å/pixel (468x468 pixel box, rescaled to 256x256 pixels) using Relion and refined to 4.4 Å, then subjected to CTF refinement and refined to 4.0 Å. A second round of 3D classification using a soft solvent mask was used to identify the best resolved particles in data set 2. Two classes (327,721 particles) showed a well-resolved GroEL complex and were selected for further processing. The selected 3D classes from the grid 1 and grid 2 data sets were combined (678,597 particles). This set of particles was re-extracted at 1.2 Å/pixel in a larger box (512x512 pixels, rescaled to 448x448 pixels) and subjected to CTF refinement and particle polishing, then imported into CryoSPARC. The set of 679k polished particles was refined to 3.3 Å, subjected to CTF refinement, then refined to 2.8 Å using local refinement in CryoSPARC. The 2.8 Å map was used to model the GroEL nucleotide binding sites during model building. However, the 2.8 Å map displayed poorly resolved density for GroEL apical domains and no density for non-native Rubisco. To identify particles with bound non-native Rubisco, we performed masked 3D classification (skipping alignments) using 4 classes and a mask that encompassed the GroEL asymmetric ring cavity. Importantly, we also increased the amplitude contrast metadata value (in the Relion .star files) of particles from 10% to 30% (mentioned briefly in ref (8)). This adjustment enabled better classification of low-resolution features such as the non-native substrate. When the amplitude contrast was set to its default value of 10%, 3D classification converged with > 99% of particles in a single class. We performed this round of 3D classification on two subsets of the data for computational efficiency. Three classes from each 3D classification job showed strong density in the asymmetric ring cavity of GroEL. We combined these classes (352,247

particles) and refined the particles to 4.0 Å. The resulting map displayed density for non-native Rubisco, but the density for the apical domains of GroEL subunits 3 and 4 was poorly resolved. We created a soft mask around the apical domains of GroEL subunits 3 and 4 and used another round of 3D classification without alignments to identify the best resolved particles. Class 1 (99,764 particles) and class 4 (102,818 particles) showed improved density for subunits 3 and 4. We refined each class separately. The two resulting reconstructions were essentially identical. We therefore merged the particles from each class (202,582 particles) and re-extracted the combined set at 1.2 Å/pixel in a larger box (512x512 pixels, rescaled to 448x448 pixels). The re-extracted particles were then subjected to CTF refinement and particle polishing, and refined to 3.3 Å. The map displayed improved density for subunits 3 and 4. Local resolution estimation and local filtering were performed using Relion. The final map was further processed using DeepEMhancer (9). The map generated by DeepEMhancer was used to better resolve the extended apical domains of GroEL subunits 2, 5, and 7.

GroEL-ADP·AlF_3-Rubisco-GroES

The image processing workflow for GroEL-ADP·AlF_3-Rubisco-GroES is summarised in **Fig. S7**. For GroEL-ADP·AlF_3-Rubisco-GroES, a total of 5,571,101 particles were extracted from 11,733 motion-corrected micrographs at a pixel size of 3.4 Å/pixel (464x464 pixel box, rescaled to 112x112 pixels) in Relion. Extracted particles were subjected to iterative 2D classification in cryoSPARC. Following 2D classification, 3,637,345 particles were imported back into Relion and refined to 6.8 Å. We split the data set into two subsets and performed 3D classification (without alignments) with a 250 Å circular mask on each subset using eight classes. GroES density varied among the 3D classes. One class from each subset showed no GroES density and resembled our reconstruction of GroEL-ADP·BeF_3. We selected classes that displayed density for GroES (even if weak) and combined them (3,083,520 particles). We then performed a single round of 3D classification into 3 classes. One poorly resolved class contained 73.6% of the particles. Analysis by 2D classification suggested that particles belonging to the poorly resolved class were almost exclusively end views and overlapping particles. We selected the class with well-resolved GroEL-GroES features (814,317 particles), re-extracted the particles at 2.0 Å/pixel (512x512 pixel box, rescaled to 224x224) and refined them to 4.0 Å. To identify particles with encapsulated Rubisco, we used a similar approach to our GroEL-ADP·BeF_3-Rubisco processing workflow. We created a mask encompassing the *cis* cavity of GroEL-GroES and performed 3D classification (skipping alignments, 30% amplitude contrast) using 6 classes on two subsets of the data. One 3D class from each subset displayed a blob-like density inside the GroEL-GroES cavity. We merged these classes (30,965 particles), re-extracted particles at 0.85 Å/pixel (448x448 pixels) and refined them to 3.7 Å. The subset of 30,965 GroEL-ADP·AlF_3-Rubisco-GroES particles was imported into cryoSPARC v.3.3.1 and subjected to a single round of 3D classification (4 classes, target resolution = 8 Å, number of O-EM epochs = 10, number of final full iterations = 5, batch size per class = 3000, initial structure lowpass resolution = 12 Å, initialisation mode = PCA, class similarity = 0) using a soft edge mask encompassing the Rubisco density. Each resulting class of particles was

refined to a resolution of 4.1 - 4.2 Å. Local resolution estimation and local filtering were performed using tools within cryoSPARC.

GroEL-ADP·AlF$_3$-Rubisco

The image processing workflow for GroEL-ADP·AlF$_3$-Rubisco is summarised in **Fig. S8**. To obtain a reconstruction of GroEL-ADP·AlF$_3$-Rubisco, we started from the GroES-free 3D classes from the initial round of 3D classification in the GroEL-ADP·AlF$_3$-Rubisco-GroES data set (**Fig. S7**) We selected two classes that lacked density for GroES (even at low thresholds) and combined them (511,151 particles, 3.4 Å/pixel). We then performed a single round of 3D classification into 4 classes. We selected one class with well-resolved GroEL features (370,167 particles), re-extracted the particles at 2.0 Å/pixel (512x512 pixel box, rescaled to 224x224) and refined them to 4.0 Å. We performed an additional round of 3D classification into 4 classes. We selected two classes with well-resolved GroEL features (280,885 particles). To identify particles with encapsulated Rubisco, we used the same approach as for our GroEL-ADP·BeF$_3$-Rubisco processing workflow. We used the same mask encompassing the *cis* cavity of GroEL and performed 3D classification (skipping alignments, 30% amplitude contrast) using 4 classes. One class displayed strong density inside the GroEL cavity. We re-extracted particles belonging to this class (44,070 particles) at 0.85 Å/pixel (448x448 pixels), imported the particles into CryoSPARC and refined them to 3.7 Å. Local resolution estimation and local filtering were then performed using tools within cryoSPARC.

Model building

For GroEL-Rubisco, we used UCSF Chimera (10) to rigid body fit the crystal structure of GroEL (PDB: 1SS8) into the locally filtered density map generated by RELION. The model was refined into the density using Isolde v.1.3 (11), followed by real space refinement using Phenix (12). Secondary structure, reference model and geometry restraints were applied during model refinement.

For GroEL-ADP·BeF$_3$-Rubisco, we used the crystal structure of apo GroEL (PDB: 1SS8) as an initial model for the four substrate-contacting GroEL subunits in the asymmetric ring (subunits 1, 3, 4, and 6). These initial models were refined in Isolde using adaptive distance restraints. We applied distance restraints individually to the three domains of each GroEL subunit as follows: equatorial domain (residues 1-33, 409-525), intermediate domain (residues 134-190), and apical domain (residues 377-408, 191-376). This allowed each GroEL domain to move as a separate restrained body. Each GroEL subunit was then refined into the density. We used the crystal structure of GroEL-GroES (PDB: 1SVT) as an initial model for GroEL subunits 2, 5, and 7 and refined these models in Isolde. For the symmetric ring, we modelled a single GroEL subunit using the crystal structure of apo GroEL (PDB: 1SS8), then copied the refined model to the other six positions. The whole symmetric ring was then refined into the density. Inter-ring and inter-subunit contacts were then refined. For nucleotide binding sites, ADP, Mg^{2+}, and K^+ were placed into the density and refined using Isolde. BeF_3 was placed into the density and refined using Coot.

The structure was then iteratively refined using Coot and phenix.real_space_refine applying secondary structure, reference model, and geometry restraints. We used three maps to interpret, build and refine different parts of the model of GroEL-ADP·BeF_3-Rubisco. First, compared to the final 3.4 Å map (202k particles), the 2.8 Å map (678k particles), determined prior to 3D classification of bound Rubisco, displayed better resolved density for GroEL nucleotide binding sites. We therefore used the 2.8 Å map (678k particles) to model ADP, BeF_3, metal ions, and waters. Second, the map generated by DeepEMhancer was used to visualise the complex and aid rigid body fitting of GroEL subunits 2, 5, and 7, this map was not used for real space refinement. Third, the locally filtered map generated by RELION was used to refine the model using Isolde and Phenix.

For GroEL-ADP·AlF_3-Rubisco-GroES, we used UCSF Chimera to rigid body fit the crystal structure of GroEL-GroES (PDB: 1SVT) into the locally filtered map generated by cryoSPARC. The model was refined into the density using Isolde v.1.3 and phenix.real_space_refine. ADP, Mg^{2+}, and K^+ were placed into the density using Isolde. AlF_3 was placed into the density and refined using Coot. Secondary structure, reference model and geometry restraints were applied during real-space refinement in Phenix. To model Rubisco we used the locally filtered map of class II, here the Rubisco density had a local resolution of approximately 9 – 12 Å. We used ChimeraX (13) to perform a rigid body fit of one Rubisco monomer (PDB: 9RUB) into the density, positioning the larger C-terminal domain (CTD) of Rubisco into the larger lobe of substrate density volume. The Rubisco monomer was flexibly fitted to the density using the Namdinator (14) web service with a map resolution of 10 Å. Further refinement of the complete model was carried out using Isolde (11) and real-space refinement in Phenix (12). Adaptive distance restraints were applied to the Rubisco monomer during refinement in Isolde.

References

1. S. Q. Zheng, *et al.*, MotionCor2 - anisotropic correction of beam-induced motion for improved cryo-electron microscopy. *Nat Methods* **14**, 331–332 (2017).

2. K. Zhang, Gctf: Real-time CTF determination and correction. *J Struct Biol* **193**, 1–12 (2016).

3. J. M. Bell, M. Chen, T. Durmaz, A. C. Fluty, S. J. Ludtke, New software tools in EMAN2 inspired by EMDatabank map challenge. *J Struct Biol* **204**, 283–290 (2018).

4. J. Zivanov, *et al.*, New tools for automated high-resolution cryo-EM structure determination in RELION-3. *eLife* **7**, e42166 (2018).

5. A. Punjani, J. L. Rubinstein, D. J. Fleet, M. A. Brubaker, cryoSPARC: algorithms for rapid unsupervised cryo-EM structure determination. *Nat Methods* **14**, 290–296 (2017).

6. D. Asarnow, E. Palovcak, Y. Cheng, asarnow/pyem: UCSF pyem v0.5 (2019) https:/doi.org/10.5281/zenodo.3576630 (March 5, 2023).

7. D. K. Clare, *et al.*, ATP-triggered conformational changes delineate substrate-binding and -folding mechanics of the GroEL chaperonin. *Cell* **149**, 113–123 (2012).

8. M. A. Herzik, M. Wu, G. C. Lander, High-resolution structure determination of sub-100 kDa complexes using conventional cryo-EM. *Nat Commun* **10**, 1032 (2019).

9. R. Sanchez-Garcia, *et al.*, DeepEMhancer: a deep learning solution for cryo-EM volume post-processing. *Commun Biol* **4**, 1–8 (2021).

10. E. F. Pettersen, *et al.*, UCSF Chimera--a visualization system for exploratory research and analysis. *J Comput Chem* **25**, 1605–1612 (2004).

11. T. I. Croll, ISOLDE: a physically realistic environment for model building into low-resolution electron-density maps. *Acta Crystallogr D Struct Biol* **74**, 519–530 (2018).

12. D. Liebschner, *et al.*, Macromolecular structure determination using X-rays, neutrons and electrons: recent developments in Phenix. *Acta Cryst D* **75**, 861–877 (2019).

13. E. F. Pettersen, *et al.*, UCSF ChimeraX: Structure visualization for researchers, educators, and developers. *Protein Sci* **30**, 70–82 (2021).

14. R. T. Kidmose, *et al.*, Namdinator - automatic molecular dynamics flexible fitting of structural models into cryo-EM and crystallography experimental maps. *IUCrJ* **6**, 526–531 (2019).

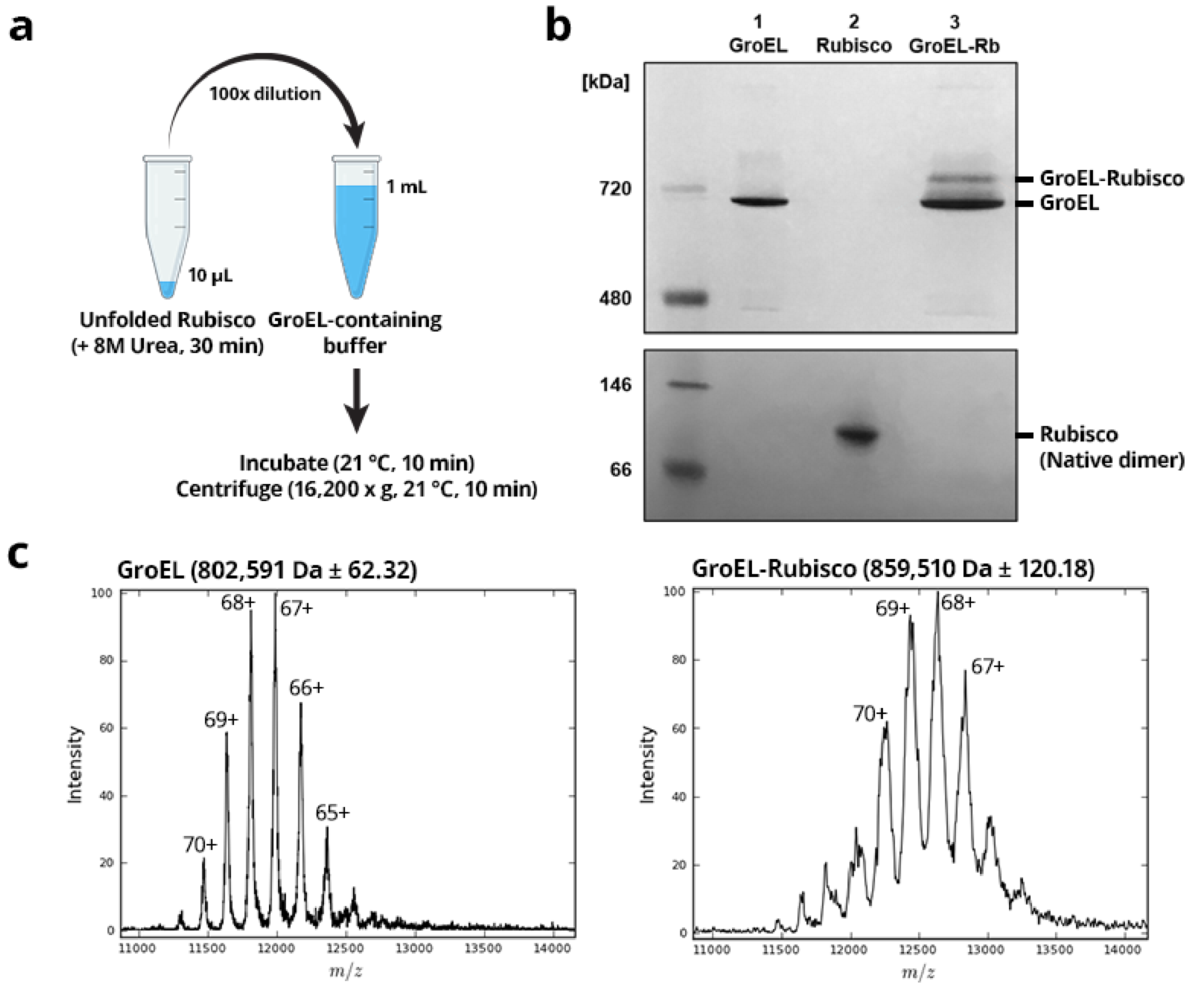

Supplementary Figure 1. Formation of GroEL-Rubisco binary complexes. (**a**) Schematic representation of the protocol to form binary complexes. (**b**) Blue Native-PAGE of purified GroEL (lane 1), purified *R. rubrum* Rubisco in its native dimeric form (lane 2), and GroEL-Rubisco complexes (lane 3). (**c**) Native electrospray ionisation mass spectrometry (ESI-MS) of purified GroEL (left) and GroEL-Rubisco complexes (right). The charge state of each prominent peak is indicated.

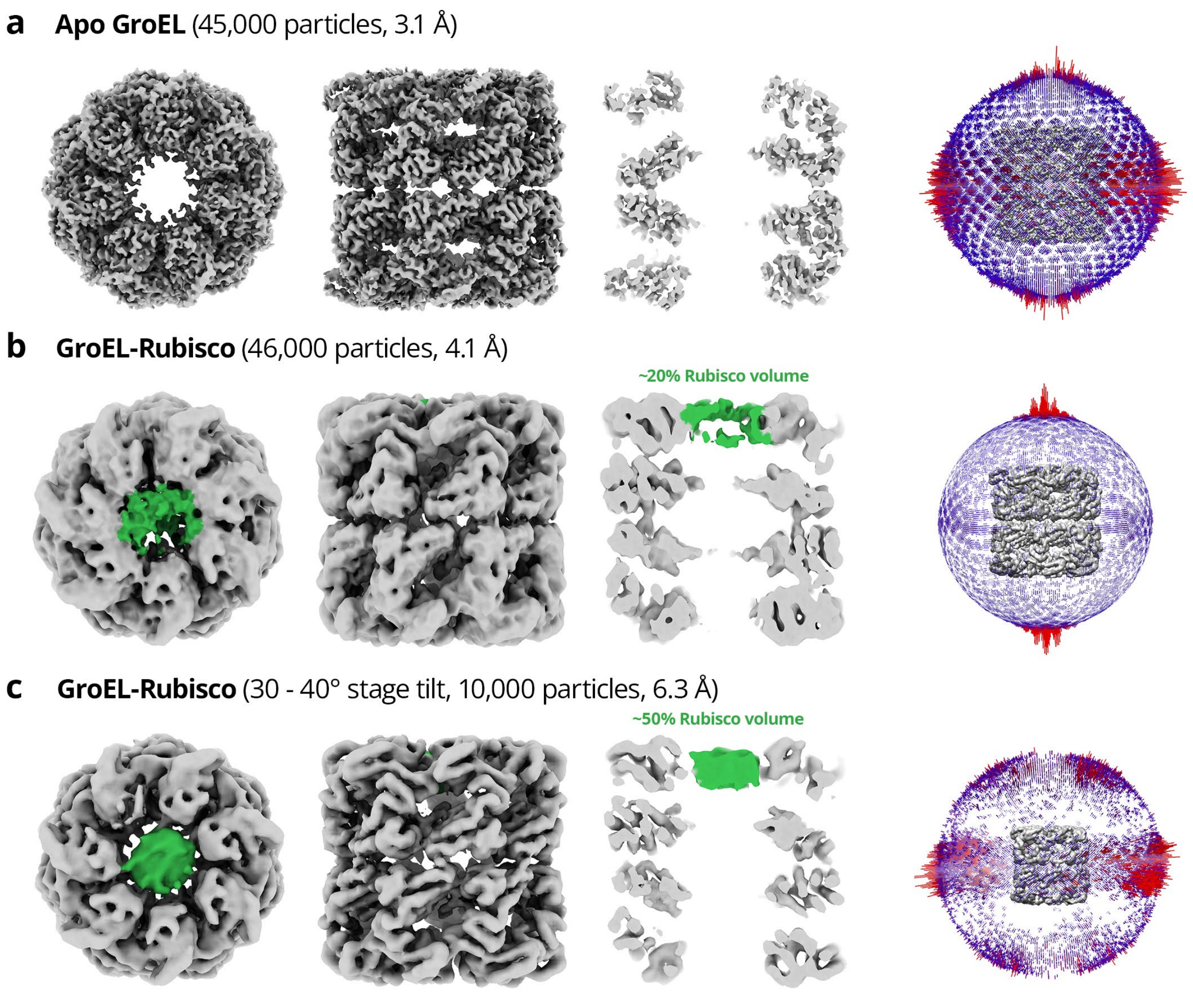

Supplementary Figure 2. Summary of initial cryoEM experiments showing the preferred orientation of GroEL-Rubisco compared to apo GroEL. Initial cryoEM data sets were collected from grids prepared using a Vitrobot. (**a**) Final reconstruction and angular distribution of apo GroEL. (**b**) Final reconstruction and angular distribution of GroEL-Rubisco. (**c**) Final reconstruction and angular distribution of GroEL-Rubisco from cryoEM data collected employing a 30 – 40° stage tilt. Density for GroEL and Rubisco is coloured grey and green respectively. For GroEL-Rubisco complexes, the Rubisco density volume is expressed as a percentage of the theoretical volume of a folded Rubisco monomer.

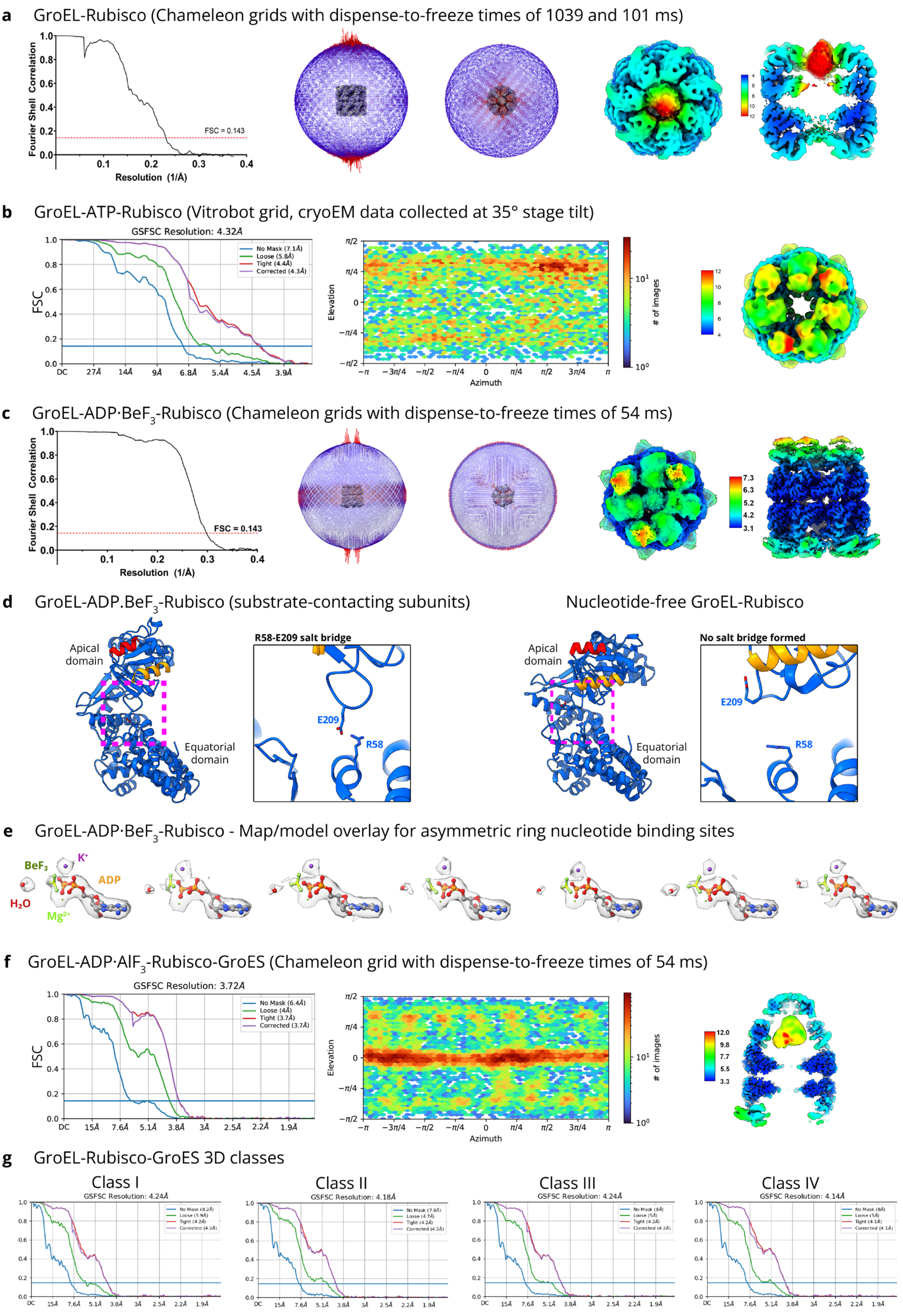

Supplementary Figure 3. CryoEM map validation information for (**a**) GroEL-Rubisco, (**b**) GroEL-ATP-Rubisco, (**c**) GroEL-ADP·BeF_3-Rubisco. (**d**) The R58-E209 salt bridge observed in

GroEL-ADP·BeF_3-Rubisco is not present in nucleotide-free GroEL-Rubisco. (**e**) Map and model overlay for nucleotide binding pockets for GroEL subunits in both rings of GroEL-ADP·BeF_3-Rubisco. CryoEM map validation information for (**f**) GroEL-ADP·AlF_3-Rubisco-GroES and (**g**) GroEL-ADP·AlF_3-Rubisco-GroES 3D class refinements.

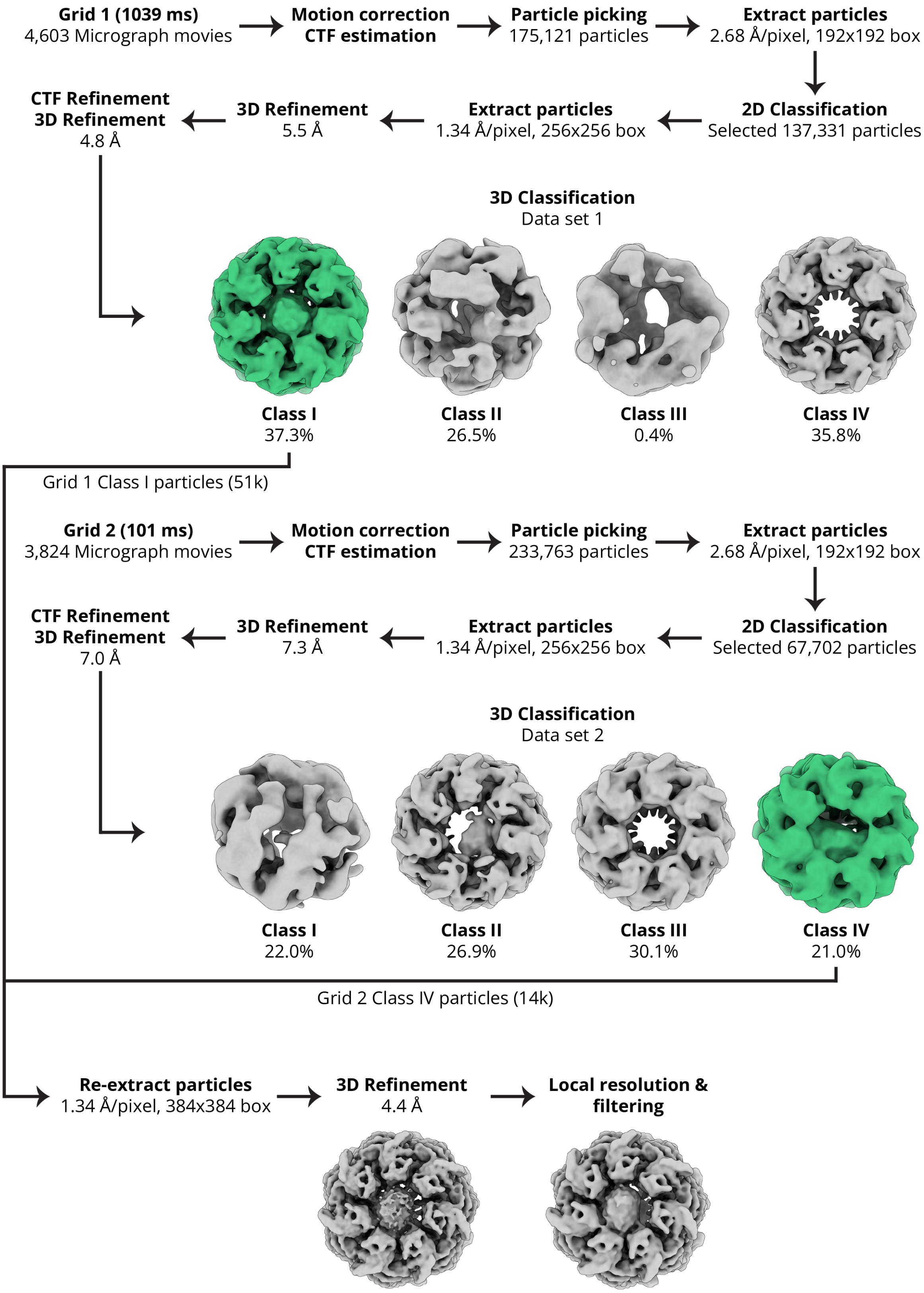

Supplementary Figure 4. CryoEM image processing workflow for GroEL-Rubisco. Processing steps were performed using Relion v.3.1. and cryoSPARC v.3.3.1 (see methods for details). Classes selected for further processing are coloured green.

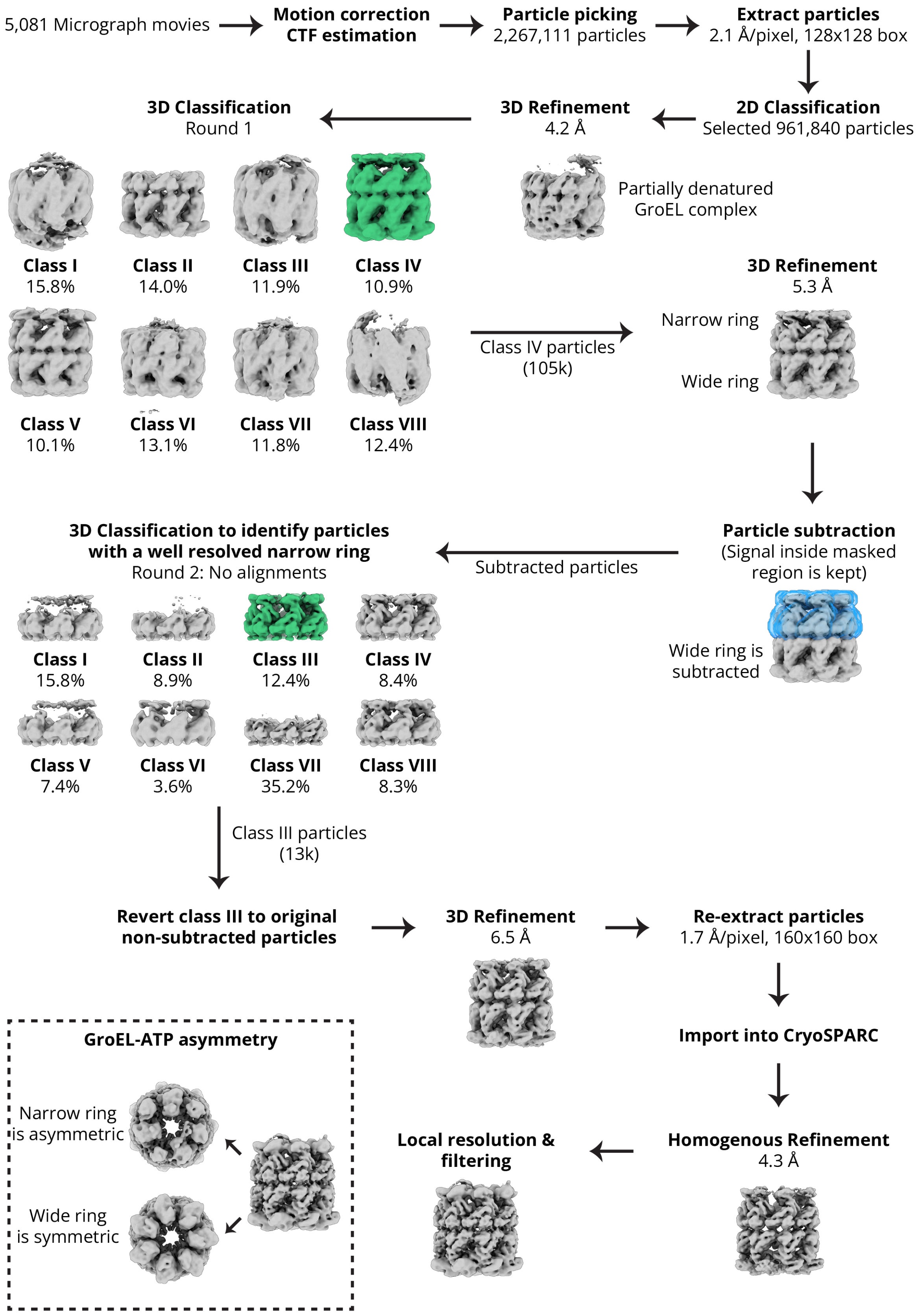

Supplementary Figure 5. CryoEM image processing workflow for GroEL-ATP-Rubisco. Processing steps were performed using Relion v.3.1. and cryoSPARC v.3.3.1 (see methods

for details). Classes selected for further processing are coloured green. Mask used during particle subtraction is coloured in blue.

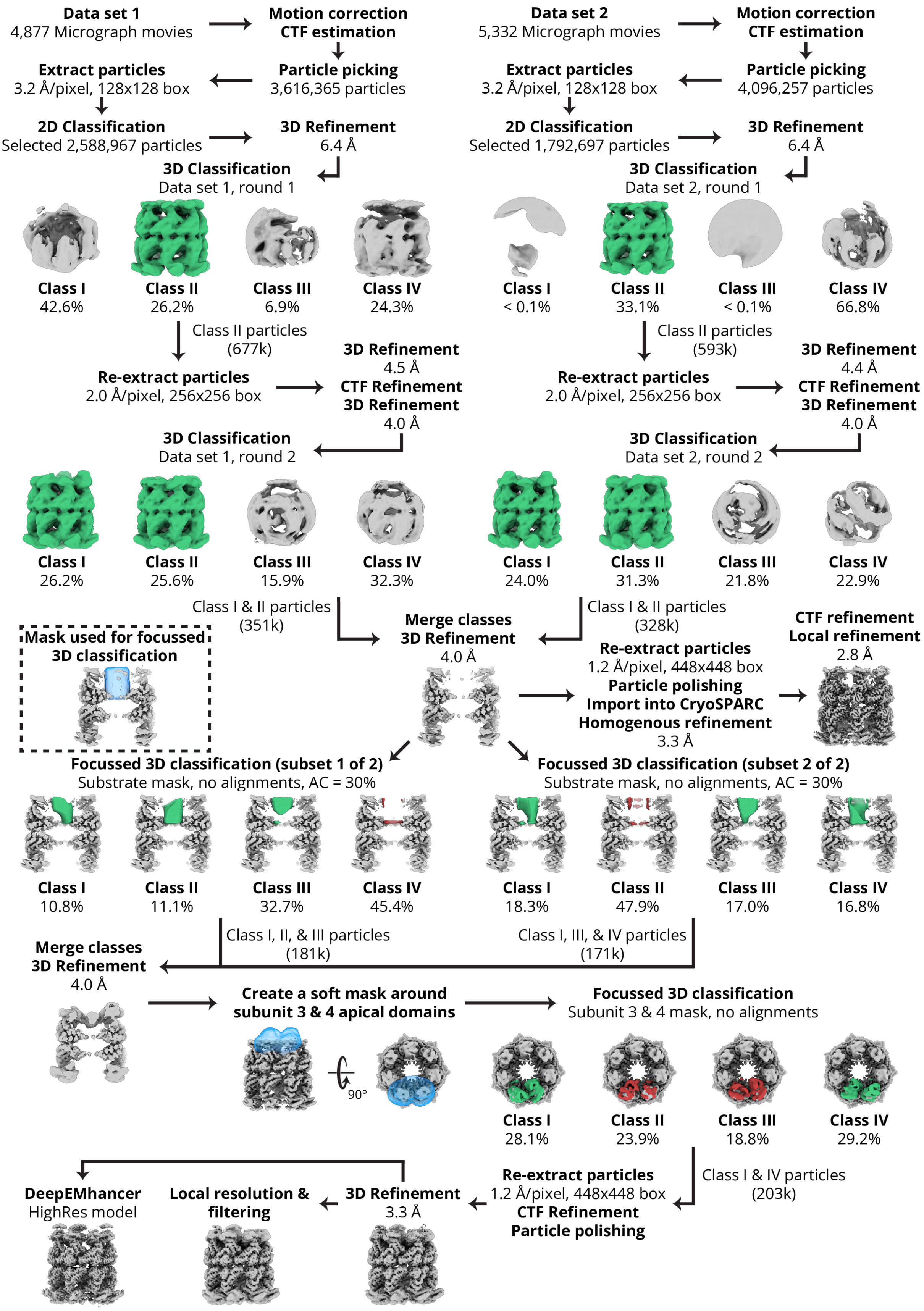

Supplementary Figure 6. CryoEM image processing workflow for GroEL-ADP·BeF_3-Rubisco. Processing steps were performed using Relion v.3.1. and cryoSPARC v.3.3.1 (see methods for details). Amplitude contrast abbreviated as AC. Classes selected for further

processing are coloured green. In focussed 3D classification jobs, discarded classes are coloured red. Masks used during focussed 3D classification are coloured in blue.

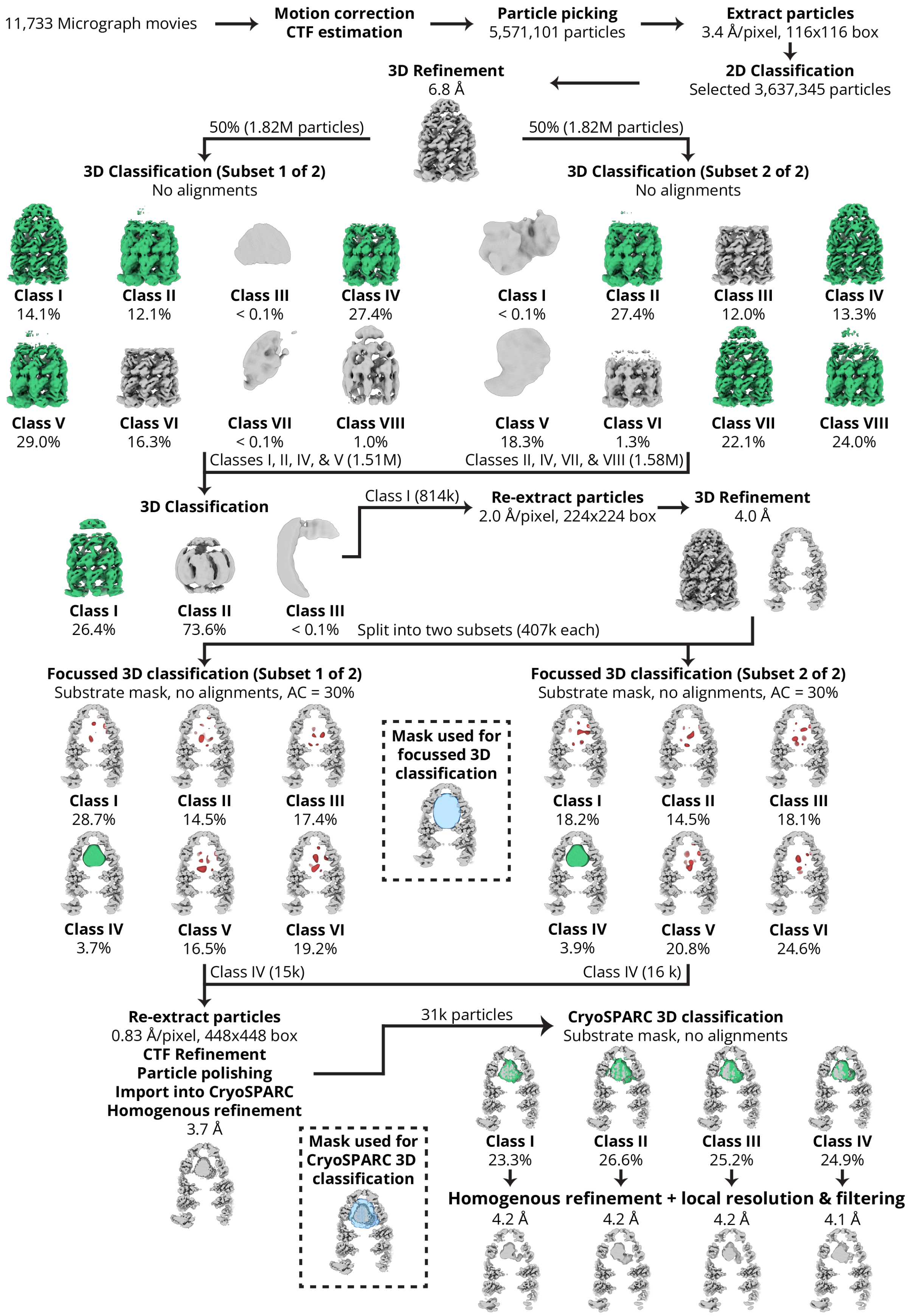

Supplementary Figure 7. CryoEM image processing workflow for GroEL-ADP·AlF_3-Rubisco-GroES. Processing steps were performed using Relion v.3.1. and cryoSPARC

v.3.3.1 (see methods for details). Amplitude contrast abbreviated as AC. Classes selected for further processing are coloured green. In focussed 3D classification jobs, discarded classes are coloured red. Masks used during focussed 3D classification are coloured in blue.

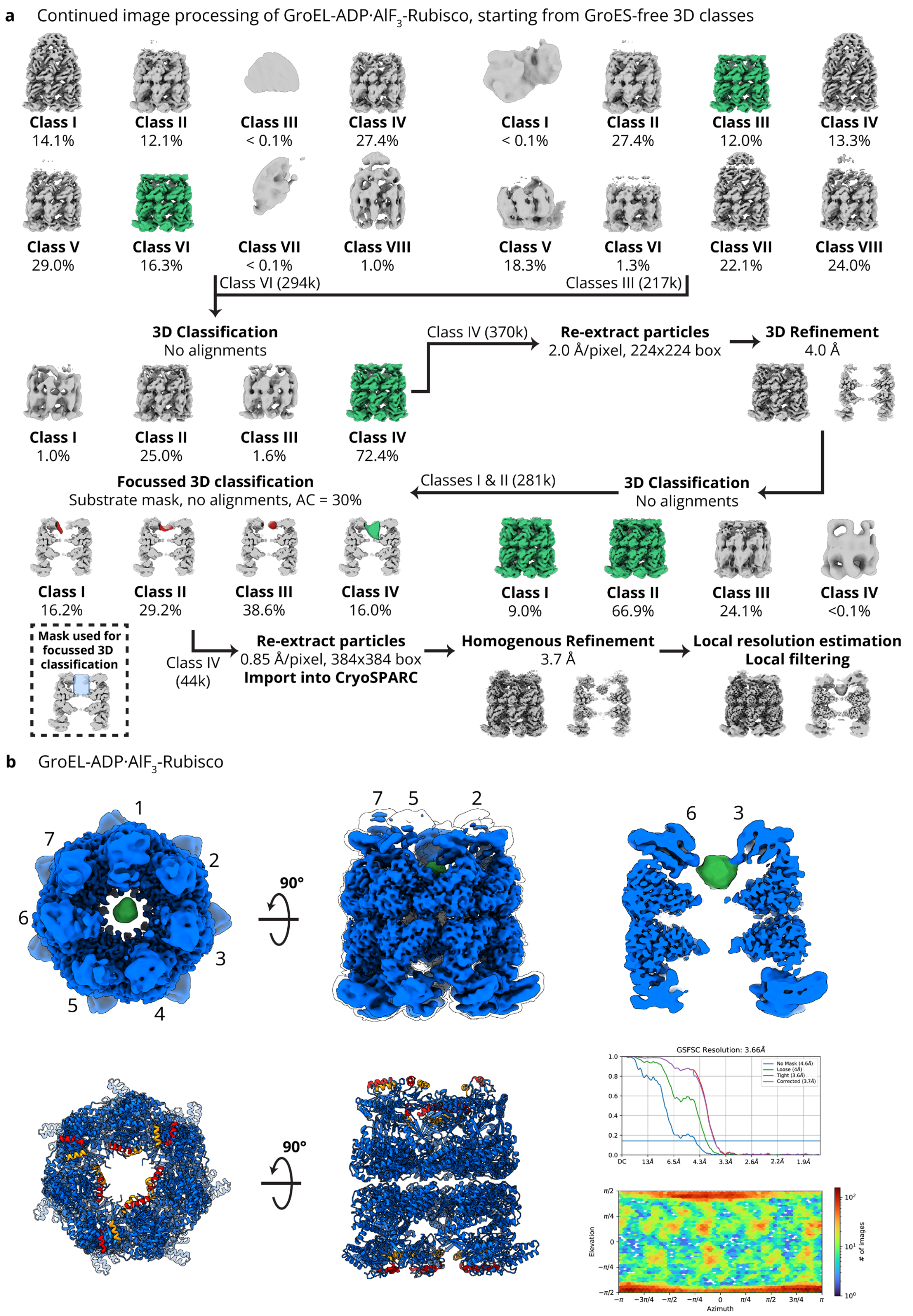

Supplementary Figure 8. (**a**) CryoEM image processing workflow for GroEL-ADP·AlF_3-Rubisco. Processing steps were performed using Relion v.3.1. and cryoSPARC v.3.3.1 (see

methods for details). Amplitude contrast abbreviated as AC. Classes selected for further processing are coloured green. In focussed 3D classification jobs, discarded classes are coloured red. The mask used during focussed 3D classification are coloured in blue. (**b**) CryoEM map, model, and validation information for GroEL-ADP·AlF_3-Rubisco. GroEL asymmetric ring subunits are numbered 1 – 7. CryoEM maps are displayed at different contour levels to show the interaction with non-native Rubisco.

Sample	GroEL-Rubisco		GroEL-Rubisco + ATP	GroEL-Rubisco + $ADP \cdot BeF_3$		GroEL-Rubisco + $ADP \cdot AlF_3$ + GroES
**EMDB**	15939		15941	15940		15944
**PDB**	8BA7		N/A	8BA8		8BAA
	Dataset 1	Dataset 2		Dataset 1	Dataset 2	
**Sample preparation**						
Protein concentration (μM)	3.4	3.4	1.0	7.0	7.0	7.0
Plunge-freeze instrument	Chameleon	Chameleon	Vitrobot	Chameleon	Chameleon	Chameleon
Dispense-to-plunge time (ms)	1039	101	N/A	54	54	54
**Data collection**						
Microscope	Titan Krios	Titan Krios	Titan Krios	Titan Krios	Titan Krios	Titan Krios
Voltage (keV)	300	300	300	300	300	300
Magnification (x1000)	64	64	130	130	130	165
Stage tilt (°)	0	0	35	0	0	0
Electron exposure (e^-/A^2)	40.2	40.2	49.1	50.0	50.0	72.3
Defocus range (μm)	1.4 – 3.0	1.4 – 3.0	0.5 – 2.7	1.5 – 2.7	1.5 – 2.7	1.5 – 2.7
Detector	K2	K2	K3	K3	K3	K3
Collection mode	Counting	Counting	Super resolution	Super resolution	Super resolution	Super resolution
Pixel size (Å)	1.340	1.340	1.06	1.068	1.068	0.85
# Micrograph movies	4603	3824	5081	4877	5332	11733
# Particles (After 2D classification)	137,331	67,702	961,840	2,588,967	1,792,697	3,637,345
# Average particles per micrograph	30	18	189	531	336	310
**Reconstruction**						
# Particles (final)	65,453		13,015	202,582		8,237
Map resolution (Å) (FSC threshold = 0.143)	4.4		4.3	3.4		4.2
Map resolution range (Å)	4.2 – 12.0			3.1 – 7.1		4.0 – 12.0
Map sharpening ($Å^2$)	-130			-60		-60
**Model refinement**						
Model resolution (Å) (FSC threshold = 0.5)	4.3			3.2		4.3
Number of residues						
Protein	7336			7336		8467
Ligand				ADP: 14<br>BeF_3: 7<br>MG: 14<br>K: 14		ADP: 14<br>AlF_3: 7<br>MG: 14<br>K: 7
Water				7		
B-factors ($Å^2$)						
Protein	162.50			174.29		223.68
Ligand				85.43		127.15
Water				85.06		
R.M.S. deviations						
Bond lengths (Å)	0.002			0.005		0.005
Bond angles (°)	0.519			1.072		0.847
**Validation**						
MolProbity score	1.83			1.79		1.95
Clashscore	12.03			9.09		13.34
Rotamer outliers (%)	0.00			0.00		0.00
Ramachandran plot						
Favoured (%)	96.43			95.65		95.49
Allowed (%)	3.57			4.35		4.45
Outliers (%)	0.00			0.00		0.06

Supplementary Table 1. Sample preparation details, cryoEM data collection parameters, map reconstruction and model refinement statistics.
